# Whole-genome sequencing identifies variants in *ANK1*, *LRRN1*, *HAS1,* and other genes and regulatory regions for stroke in type 1 diabetes

**DOI:** 10.1038/s41598-024-61840-7

**Published:** 2024-06-11

**Authors:** Anni A. Antikainen, Jani K. Haukka, Anmol Kumar, Anna Syreeni, Stefanie Hägg-Holmberg, Anni Ylinen, Elina Kilpeläinen, Anastasia Kytölä, Aarno Palotie, Jukka Putaala, Lena M. Thorn, Valma Harjutsalo, Per-Henrik Groop, Niina Sandholm, the FinnDiane Study Group, Anni A. Antikainen, Jani K. Haukka, Anmol Kumar, Anna Syreeni, Stefanie Hägg-Holmberg, Anni Ylinen, Jukka Putaala, Lena M. Thorn, Valma Harjutsalo, Per-Henrik Groop, Niina Sandholm

**Affiliations:** 1 Folkhälsan Institute of Genetics, Folkhälsan Research Center, Helsinki, Finland; 2 Department of Nephrology, University of Helsinki and Helsinki University Hospital, Helsinki, Finland; 3 Research Program for Clinical and Molecular Metabolism, Faculty of Medicine, University of Helsinki, Helsinki, Finland; 4 Institute for Molecular Medicine Finland (FIMM), HiLIFE, University of Helsinki, Helsinki, Finland; 5 Analytic and Translational Genetics Unit, Department of Medicine, Department of Neurology and Department of Psychiatry, Massachusetts General Hospital, Boston, MA USA; 6 The Stanley Center for Psychiatric Research and Program in Medical and Population Genetics, The Broad Institute of MIT and Harvard, Cambridge, MA USA; 7 Neurology, Helsinki University Hospital and University of Helsinki, Helsinki, Finland; 8 Department of General Practice and Primary Health Care, University of Helsinki and Helsinki University Hospital, Helsinki, Finland; 9 Department of Diabetes, Central Clinical School, Monash University, Melbourne, VIC Australia

**Keywords:** Endocrine system and metabolic diseases, Stroke, Clinical genetics, Genetic association study

## Abstract

Individuals with type 1 diabetes (T1D) carry a markedly increased risk of stroke, with distinct clinical and neuroimaging characteristics as compared to those without diabetes. Using whole-exome or whole-genome sequencing of 1,051 individuals with T1D, we aimed to find rare and low-frequency genomic variants associated with stroke in T1D. We analysed the genome comprehensively with single-variant analyses, gene aggregate analyses, and aggregate analyses on genomic windows, enhancers and promoters. In addition, we attempted replication in T1D using a genome-wide association study (N = 3,945) and direct genotyping (N = 3,263), and in the general population from the large-scale population-wide FinnGen project and UK Biobank summary statistics. We identified a rare missense variant on *SREBF1* exome-wide significantly associated with stroke (rs114001633, p.Pro227Leu, *p*-value = 7.30 × 10^–8^), which replicated for hemorrhagic stroke in T1D. Using gene aggregate analysis, we identified exome-wide significant genes: *ANK1* and *LRRN1* displayed replication evidence in T1D, and *LRRN1*, *HAS1* and *UACA* in the general population (UK Biobank). Furthermore, we performed sliding-window analyses and identified 14 genome-wide significant windows for stroke on 4q33-34.1, of which two replicated in T1D, and a suggestive genomic window on *LINC01500*, which replicated in T1D. Finally, we identified a suggestively stroke-associated *TRPM2-AS* promoter (*p*-value = 5.78 × 10^–6^) with borderline significant replication in T1D, which we validated with an in vitro cell-based assay. Due to the rarity of the identified genetic variants, future replication of the genomic regions represented here is required with sequencing of individuals with T1D. Nevertheless, we here report the first genome-wide analysis on stroke in individuals with diabetes.

## Introduction

Stroke is a notable cause of mortality and long-term disability worldwide, with diabetes among the most important risk factors. The standardized incidence ratio is roughly sixfold among individuals with type 1 diabetes (T1D) compared to the general population^1^. Furthermore, 537 million adults live with diabetes today and the prevalence is rising^2^. Even though much of this trend is driven by an increase in obesity and insulin-resistant type 2 diabetes (T2D), the incidence of insulin-dependent T1D has increased as well^3^. T1D is a lifelong condition caused by an autoimmune reaction towards the pancreas and treated with daily insulin injections. The strokes themselves may be of hemorrhagic (20%) or ischemic (80%) origin and classified into even more specific subtypes. Interestingly, the two diabetes types affect stroke risk differentially: T1D increases the risk of both ischemic- and hemorrhagic stroke^4,5^, while the risk imposed by T2D has been estimated more modest for hemorrhagic strokes^5^. Importantly, T1D predisposes individuals to cerebral small-vessel disease and strokes of microvascular origin^6,7^. Diabetes causes also other complications, of which diabetic kidney disease (DKD) and severe retinopathy predict cerebrovascular disease in T1D^8^. Understanding stroke pathophysiology in diabetes is important for improving treatment and quality of life for individuals with T1D.

Stroke heritability has been estimated to vary between 30 and 40% in the general population^9^. Stroke heritability varies greatly depending on the subtype, with the largest heritability estimates for large artery atherosclerotic stroke and lobar intracranial hemorrhage, and the lowest for small vessel disease^9^. To date, 126 common genomic loci have been associated with stroke or its subtypes with genome-wide significance^10,11^. Associations at many of the known common stroke loci overlap with other cardiovascular phenotypes, e.g., coronary artery disease (CAD)^9^. Our previous study suggested a heritable component of stroke in individuals with T1D as a history of maternal stroke was associated with hemorrhagic stroke in T1D^12^. However, very few studies have investigated genetic risk factors for stroke in diabetes^13–15^, and no genome-wide studies in individuals with diabetes yet exist. On the other hand, genetic studies on CAD in diabetes have identified a few diabetes-specific loci^16,17^, although still pending external replication, and have replicated three known general population CAD risk loci in diabetes: *CDKN2B-AS1*, *PSRC1* and *LPA*^15,16,18^.

A substantial proportion of heritability remains unexplained for stroke^9^. Rare genetic variants with minor allele frequency (MAF) of ≤ 1% may significantly contribute to stroke heritability. In fact, some rare monogenic disorders have stroke as one of their manifestations^9,10,19^. In GWASs, the imputation accuracy of rare variants may be limited, and largely depends on the minor allele count (MAC) in the reference sample^20^. Rare variants can be reliably studied with next-generation sequencing-based techniques such as whole-genome sequencing (WGS) and whole-exome sequencing (WES). We have previously used WES to identify protein coding variants associated with lipid and apolipoprotein traits in T1D^21^. In the general population, novel stroke risk loci have been identified with WGS^22^. However, UK Biobank WES analysis for cardiometabolic traits did not discover exome-wide significant stroke risk genes^23^.

Historically, the Finnish population has been isolated and, thus, represents a unique genetic background with enrichment of low-frequency deleterious variants^24^, which may in part enable the discovery of rare disease-associated variants. Here we studied genetics of stroke and its subtypes with WGS and WES in Finnish individuals with T1D with multiple statistical approaches by focusing on rare and low-frequency genomic variants. We aimed both to find stroke-risk loci specific to individuals with T1D, and to identify risk loci generalizable to the non-diabetic population, since discovery of rare variants is more probable in a high-risk Finnish diabetic population. Finally, we performed cell-based in vitro experiments to further validate a discovered promoter region. Altogether, here we report the first genome-wide study on stroke genetics in diabetes.

## Results

### Study design

**Figure 1:**
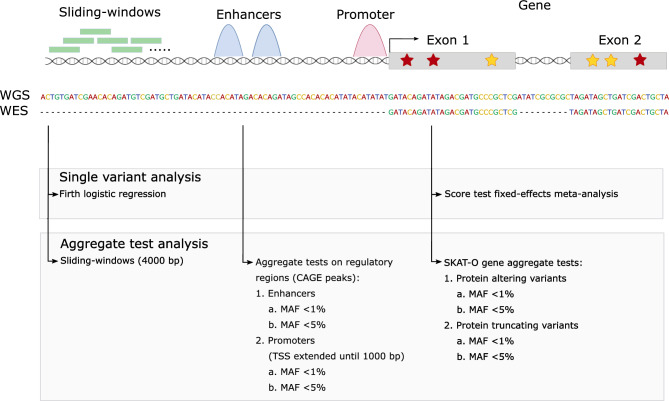
Study design.

The study is part of the Finnish Diabetic Nephropathy (FinnDiane) Study; an ongoing nationwide multicenter study established to identify factors leading to diabetic complications^25^. We studied WGS in 571 and WES in 480 non-related and non-overlapping individuals with T1D, entailing 112 and 74 stroke cases, respectively (Table 1, Table S1, Fig. S1 and S2). We aimed to find rare and low-frequency genetic variants associated with stroke in T1D. Therefore, we performed single variant analyses across the genome (MAC ≥ 5), using fixed-effects meta-analysis for variants available in both data sets, with a minimal adjustment setting i.e., the calendar year of diabetes onset, sex and two first genomic data principal components, and repeated the analyses with an additional DKD adjustment (Fig. 1). We performed gene aggregate analyses (cumulative MAC, CMAC ≥ 5) with the minimal adjustment separately with protein-altering variants (PAVs) and protein-truncating variants (PTVs); and repeated the analyses with an additional DKD adjustment. Finally, we conducted minimally adjusted intergenic aggregate analyses within genomic windows by statistically up-weighting functionally important and rare variants; and within established enhancers and promoters by weighting variants according to their rarity. Furthermore, we performed stroke subtype association analyses for the lead findings.

**Table 1:** Clinical characteristics of study participants in the next-generation sequencing data sets.

	WES			WGS		
	Cases	Controls	*p*-value	Cases	Controls	*p*-value
N	74	406		112	459	
Hemorrhagic/ischemic	22/49			26/64		
CVD death < 2017 (yes/no, % yes)	35/39 (47%)	66/340 (16%)	2.92 × 10^–8^	58/54 (52%)	83/376 (18%)	2.61 × 10^–12^
Sex (male/female, % males)	39/35 (53%)	182/224 (45%)	0.25	80/32 (71%)	236/223 (51%)	0.00013
Age (years)	48.39 (11.89)	60.69 (11.13)	7.08 × 10^–13^	51.08 (10.29)	58.70 (9.61)	3.52 × 10^–11^
T1D duration (years)	33.6 (10.45)	47.49 (9.84)	5.89 × 10^–18^	36.16 (9.81)	46.00 (8.14)	6.34 × 10^–18^
Calendar year of T1D onset*	1969.5 (11.75)	1968 (11)	0.29	1968 (10.25)	1969 (9)	0.80
T1D onset age (years)	14.79 (6.64)	13.21 (7.27)	0.066	14.92 (8.93)	12.69 (7.75)	0.016
Weighted mean HbA_1c_ (%)*	9.00 (2)	8.51 (1.4)	0.0037	8.86 (1.76)	8.34 (1.57)	0.00097
HbA_1c_ count*	19 (28.5)	29 (26)	0.0038	19 (30)	29 (30)	0.011
DKD (yes/no, yes-%)	52/22 (70%)	201/205 (50%)	0.00098	87/25 (78%)	206/253 (45%)	2.49 × 10^–10^

Weighted mean HbA1c is calculated until the stroke event or the end of follow-up. DKD: diabetic kidney disease status closest to the end of follow-up, defined as end-stage renal disease (ESRD), macro- or microalbuminuria. Mean (standard deviation, SD), *Median (interquartile range, IQR). Student’s t-test, Wilcoxon signed rank test or Fisher’s exact test.

### Single variant analyses

**Figure 2:**
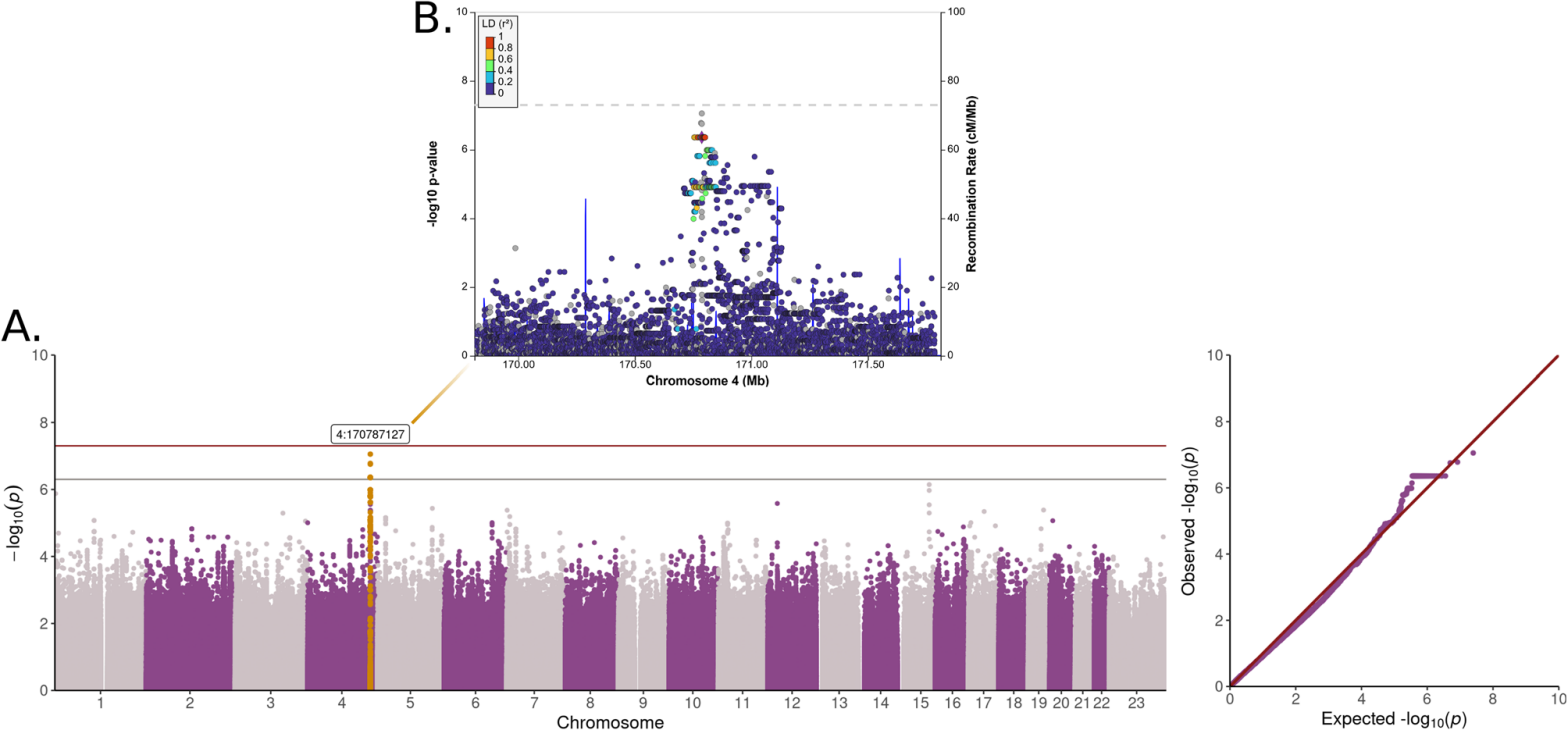
Additive single variant analysis with minimal adjustment. (**A**) Manhattan plot, (**B**) LocusZoom^60^ plot of 4q33.1-34 (LD structure according to a nearby variant, rs4386563).

We sought for genetic variants associated with stroke using non-overlapping WES and WGS data, and discovered a suggestively stroke-associated locus, 4q33-34.1, with the minimally adjusted model (4:170787127, *p*-value = 8.83 × 10^–8^, MAF = 3.7%, Table 2, Fig. 2). The variant was unavailable for replication in the T1D specific GWAS and in the FinnGen general population GWAS summary statistics. However, the variant with the third lowest *p*-value on 4q33-34.1 was available but did not replicate for stroke in T1D nor the general population (Table 2). As DKD is a common diabetic complication that has been reported to predict incident stroke in T1D^8^, we performed additional analyses adjusted for DKD, and discovered a rare missense variant on *SREBF1* exome-wide significantly (*p*-value < 3 × 10^–7^) associated with stroke (rs114001633, p.Pro227Leu*, p*-value = 7.30 × 10^–8^, MAF = 0.26%) (Table 2, Fig. S3). Due to the rarity of the variant, we performed additional genotyping for replication, whereby the variant did not replicate for stroke (Table 2), but replicated for hemorrhagic stroke in T1D (*p*-value = 0.02, N = 3,263, Table S2). Since rs114001633 did not pass MAC threshold in the hemorrhagic stroke sub-analysis of the discovery cohort (Table S2), further replication in additional individuals with T1D is needed to confirm the potential association with stroke, specifically with hemorrhagic stroke, in T1D.

**Table 2:** Lead variants discovered with single variant association analyses (*p*-value < 5 × 10^–7^).

	SNP ID	Position	Potential target gene	Data	REF/ALT	MAF	OR [95% CI]	I^2^	*p*-value	FinnDiane OR (*p*-value)	FinnGen OR (*p*-value)
**Stroke** Minimal adjustment	rs4435704	4:170787127	*AADAT, MFAP3L, GALNTL6*	WGS	T/C	3.7%	6.04 [3.12–11.67]	–	8.83 × 10^–8^	–	–
	rs376936219	4:170784011		WGS	G/T	4.6%	4.79 [2.66–8.60]	–	1.67 × 10^–7^	0.88 (0.35)	1.03 (0.34)
	rs4401420	4:170787143		WGS	C/T	4.1%	5.16 [2.79–9.55]	–	1.76 × 10^–7^	–	–
**Stroke** DKD adjustment	rs114001633	17:17819659	*SREBF1* (p.Pro227Leu)	+ +	G/A	0.3%	3804.35 [189.15–76,515.45]	35%	7.30 × 10^–8^	2.26 (0.30)	1.03 (0.87)

Two variants closest to rs4435704 are reported from the 4q33-34.1 locus. In addition, if the variant was discovered already in the minimal model, we do not report DKD adjusted results. Variants are in Hardy–Weinberg equilibrium (p-value > 0.05). FinnDiane replication is performed with GWAS for rs376936219, and with genotyping for rs114001633. +  + Meta-analysis with positive minor allele effect direction, REF = Reference allele, ALT = Alternative allele, I^2^ = Meta-analysis heterogeneity estimate, OR = Odds ratio, CI = confidence interval.

### Gene aggregate analyses

**Figure 3:**
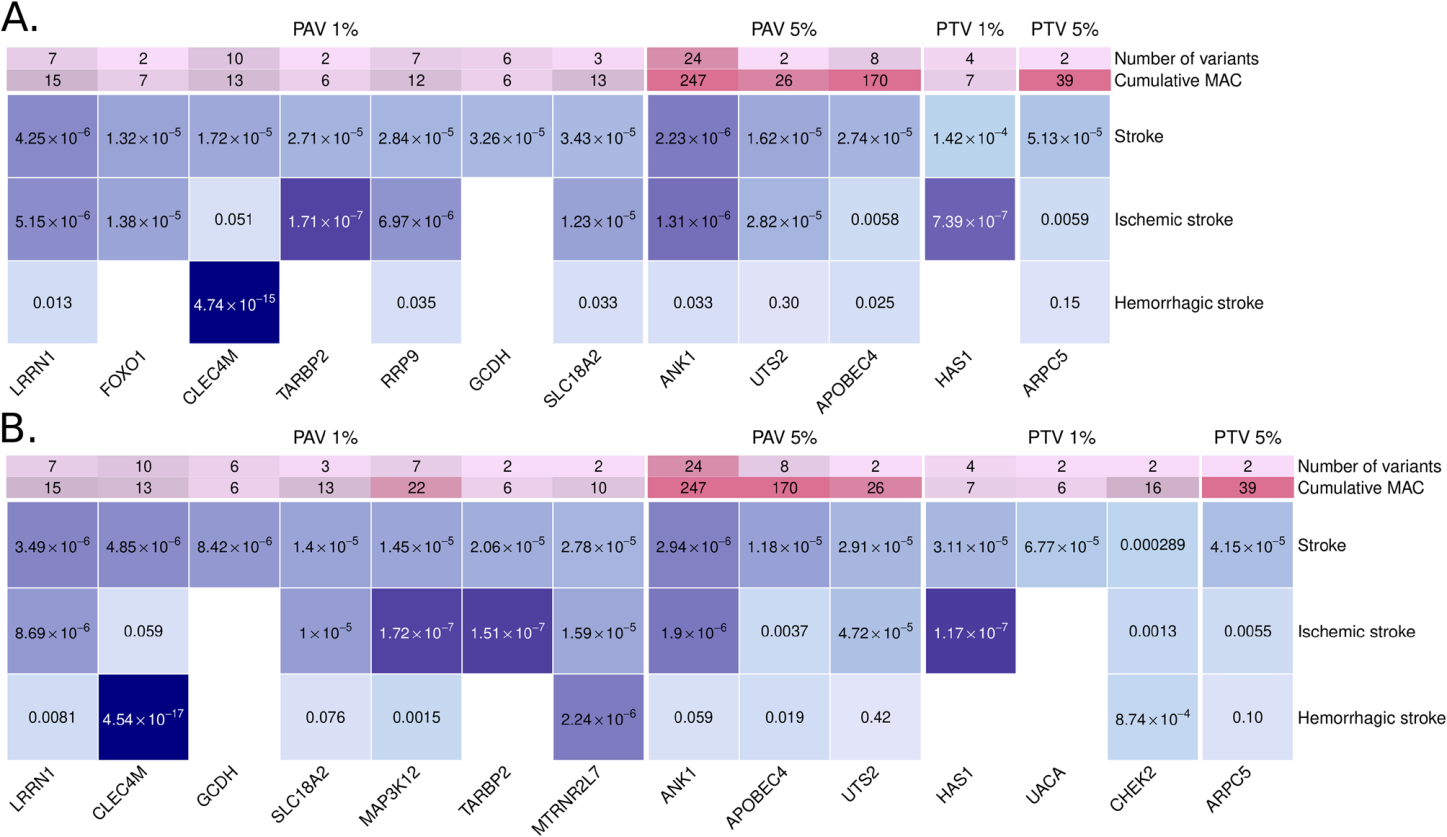
Discovered genes with the SKAT-O gene aggregate tests. (**A**) Minimal adjustment, (**B**) Additional adjustment for diabetic kidney disease. The color indicates the -log_10_(*p*-value), with darker color indicating more significant finding. Only the rare variant model (MAF ≤ 1%) is reported, if no low-frequency variants (1% < MAF ≤ 5%) were available in the gene. Bonferroni corrected significance thresholds: 4 × 10^–6^ (protein altering variant, PAV ≤ 1%), 4 × 10^–6^ (PAV ≤ 5%), 6 × 10^–5^ (protein truncating variant, PTV ≤ 5%), and 8 × 10^–5^ (PTV ≤ 1%). Number of variants and CMAC given based on the combined stroke phenotype.

To improve statistical power for rare and low-frequency variants, we performed gene aggregate analyses. With the minimally adjusted models, low-frequency PAVs on *ANK1* were associated with stroke (*p*-value = 2.23 × 10^–6^, CMAC = 247), even more strongly with ischemic stroke (*p*-value = 1.31 × 10^–6^, CMAC = 225) (Fig. 3A, Fig. S4, Tables 3, S3 and S4). Furthermore, nine genes were suggestively associated with stroke through rare or low-frequency PAVs (Fig. 3A). Of these, the aggregate of PAVs on *TARBP2* was associated with ischemic stroke (*p*-value = 1.71 × 10^–7^, CMAC = 5, MAF ≤ 1%), and on *CLEC4M* with hemorrhagic stroke (*p*-value = 4.74 × 10^–15^, CMAC = 11, MAF ≤ 1%). Of note, rare PAVs on *GCDH* were suggestively associated with stroke (*p*-value = 3.26 × 10^–5^, CMAC = 6): *GCDH* loss-of-function variants have been previously associated with metabolic stroke and cerebral hemorrhage^26^.

**Table 3:** Variants in *ANK1*, *LRRN1*, *HAS1*, and *UACA* (SKAT-O).

				Sequencing			FinnDiane GWAS		FinnGen GWAS	
Gene (model)	SNP ID	Consequence (SIFT/PolyPhen)	REF/ALT	MAC	OR [95% CI]	*p*-value	MAC	OR (*p*-value)	MAC	OR (*p*-value)
*HAS1* (Minimal + DKD: PTV ≤ 1%)	19:51713664	p.Tyr500* (–/–)	G/T	–/ ≤ 3	0.30 [0.0059–15.03]	0.54				
	19:51713853	p.Cys437* (–/–)	G/T	–/ ≤ 3	0.30 [0.0013–66.66]	0.66				
	rs1396240967	Splice donor (–/–)	C/T	–/ ≤ 3	3944.19 [9.61–1,618,806.97]	0.0070				
	rs58597876	Splice donor (–/–)	C/CACACACACACA	≤ 3/–	1152.86 [44.54–29,839.85]	2.23 × 10^–5^				
*LRRN1* (Minimal + DKD: PAV ≤ 1%)	rs755350940	5 prime UTR (–/–)	C/T	≤ 3/ ≤ 3	4.44 [0.17–114.85]	0.37	20	1.05 (0.93)	1483	0.98 (0.85)
	rs142381203	p.Arg185Cys (0.00/0.79)	C/T	≤ 3/–	67.36 [0.67–6741.25]	0.074	≤ 3	0.82 (0.46**)		
	rs147007969	p.Thr190Ile (0.18/0.54)	C/T	≤ 3/4	33.45 [3.87–288.88]	0.0014	25	0.75 (0.61)	1431	0.94 (0.57)
	rs141825989	p.Arg305Cys (0.01/0.80)	C/T	–/ ≤ 3	437.03 [2.16–88,574.56]	0.025	≤ 3	0.52 (0.82*)		
	rs770349026	p.Arg390His (0.00/0.98)	G/A	–/ ≤ 3	28.22 [0.44–1799.38]	0.12				
	rs747455683	p.Thr442Met (0.12/0.65)	C/T	≤ 3/–	2344.9 [6.68–822,743.69]	0.0094			281	0.75 (0.35)
	rs543878366	p.Glu481Lys (0.01/0.27)	G/A	–/ ≤ 3	0.33 [0.00049–218.59]	0.74				
*ANK1* (Minimal: PAV ≤ 5%)	rs750580242	p.Ser896Arg (0.40/0.11)	A/T	≤ 3/–	24.05 [0.35–1658.39]	0.14				
	rs751965455	p.Met1607Ile (0.06/0.07)	C/T	–/ ≤ 3	0.29 [0.0017–49.17]	0.64				
	rs146416859	p.Arg1577Cys (0.03/0.01)	G/A	≤ 3/ ≤ 3	6.17 [0.20–194.34]	0.30	15	1.66 (0.49)	716	1.01 (0.94)
	rs34664882	p.Ala114Val (0.04/0.74)	G/A	13/15	3.16 [1.14–8.75]	0.028	141	1.12 (0.63)	8250	0.93 (0.17)
	rs199760447	p.Val1450Ile (1.00/0.00)	C/T	–/ ≤ 3	5218.68 [11.53–2,362,426.91]	0.0060	6	0.26 (0.24)	257	1.12 (0.70)
	rs201439151	p.Tyr1386His (0.01/0.50)	A/G	≤ 3/ ≤ 3	0.28 [0.016–4.63]	0.37	11	0.73 (0.71)	917	0.76 (0.062)
	rs10093583	p.Met159Val (0.40/0.00)	T/C	4/ ≤ 3	2.61 [0.33–20.86]	0.36	41	0.80 (0.61)	2757	1.03 (0.72)
	rs779805849	p.Val136Glu (0.00/0.99)	A/T	≤ 3/–	3.49 [0.15–78.76]	0.43	5	24.19 (0.017)	684	0.85 (0.34)
	rs148275567	p.Arg89Cys (0.00/1.00)	G/A	≤ 3/4	8.50 [1.37–52.60]	0.022	11	0.98 (0.98)	1056	0.85 (0.23)
	rs117516263	Structural interaction (–/–)	G/A	19/12	0.87 [0.35–2.18]	0.77	114	1.39 (0.22)	6980	0.92 (0.11)
	rs202226361	p.Val1048Met (0.00/1.00)	C/T	5/–	0.84 [0.10–7.07]	0.87	21	3.30 (0.055)	1510	1.01 (0.97)
	rs767717861	p.Thr1022Met (0.00/0.93)	G/A	–/ ≤ 3	11,731.12 [20.48–6,718,664.78]	0.0038				
	8:41,696,385	p.Pro302Thr (0.00/1.00)	G/T	–/ ≤ 3	0.30 [0.012–7.42]	0.46				
	8:41,696,393	p.Ala299Glu (1.00/0.91)	G/T	–/ ≤ 3	6.75 [0.21–212.64]	0.28				
	rs2304877	p.Arg619His (0.01/0.003)	C/T	32/41	1.35 [0.72–2.53]	0.35	317	1.00 (0.98)	20,065	1.00 (0.91)
	rs61753679	Structural interaction (–/–)	G/T	20/22	0.91 [0.40–2.08]	0.84	120	1.21 (0.47)	8343	0.99 (0.79)
	rs34387324	Structural interaction (–/–)	G/A	≤ 3/ ≤ 3	0.30 [0.012–7.35]	0.46	10	1.10 (0.92)	1206	1.19 (0.18)
	rs142690258	p.Asn528Ser (0.84/0.001)	T/C	7/5	4.26 [1.00–18.18]	0.051	21	0.69 (0.55)	2327	0.97 (0.72)
	rs140085544	p.Val463Ile (0.04/0.38)	C/T	≤ 3/ ≤ 3	144.03 [4.66–4447.07]	0.0046	17	2.43 (0.20)	1101	0.93 (0.60)
	rs769619019	p.Met476Leu (0.11/0.99)	T/A	–/ ≤ 3	0.30 [0.0015–58.11]	0.65				
	rs1015648649	p.Ala461Val (0.00/1.00)	G/A	≤ 3/–	0.31 [0.0010–94.94]	0.69				
	8:41,717,683	p.His442Arg (0.00/1.00)	T/C	≤ 3/–	0.31 [0.00099–99.72]	0.69				
	rs1364511169	p.His416Arg (1.00/1.00)	T/C	≤ 3/–	0.27 [0.0025–29.50]	0.59				
	rs61735313	p.Asn251Lys (0.01/1.00)	G/T	11/4	4.10 [1.08–15.53]	0.038	77	0.82 (0.53)	3998	0.99 (0.85)
*UACA* (Minimal + DKD: PTV ≤ 1%)	rs781623644	p.Gln1116* (–/–)	G/A	≤ 3/ ≤ 3	154.47 [9.19–2597.75]	0.00045	7	0.51 (0.57)	301	1.10 (0.72)
	rs185763236	Splice donor (–/–)	C/A	≤ 3/–	235.10 [1.47–37,661.74]	0.035	≤ 3	4084.69 (0.0030*)		

*Genotyped, **Genotyped and tested without kinship adjustment. Consequence: Amino acid alteration matching to the SIFT score, SIFT = 0–1 (deleterious-tolerated), PolyPhen = 0–1 (bening-damaging); if multiple transcript alterations, we report the most severe consequence score, i.e., PolyPhen score may not match consequence. MAC = MAC in WGS / MAC in WES, REF = Reference allele, ALT = Alternative allele, OR = Odds ratio, CI = confidence interval.

With the models additionally adjusted for DKD, rare PAVs on *LRRN1* were associated with stroke (*p*-value = 3.49 × 10^–6^, CMAC = 15), and suggestively with ischemic stroke (*p*-value = 8.69 × 10^–6^, CMAC = 12; Fig. 3B, Fig. S5, Tables 3, S3 and S5). Furthermore, eight genes were suggestively associated with stroke through rare or low-frequency PAVs (Fig. 3B). In the stroke subtype analysis for the lead genes, the aggregate of rare PAVs on *MAP3K12* was associated with ischemic stroke (*p*-value = 1.72 × 10^–7^, CMAC = 17), and on *MTRNR2L7* with hemorrhagic stroke (*p*-value = 2.24 × 10^–6^, CMAC = 6). *MAP3K12* and *TARBP2* are located close to each other on the genome, thus, they may represent the same association signal through linkage disequilibrium (LD) or modifier effects onto the causal gene (Fig. S6).

We then investigated the role of more severe PTVs, i.e. putative loss-of-function variants, for stroke. Low-frequency PTVs on *ARPC5* were associated with stroke, while rare PTVs on *HAS1* (i.e., hyaluronan synthase 1) were suggestively associated with stroke (Fig. 3A, Fig. S4, Tables 3 and S4). Furthermore, in the analysis for stroke subtypes, the aggregate of rare PTVs on *HAS1* was associated with ischemic stroke (*p*-value = 7.39 × 10^–7^, CMAC = 7). With the additional DKD adjustment, rare PTVs on *HAS1* (*p*-value = 3.11 × 10^–5^, CMAC = 7), rare PTVs on *UACA* (*p*-value = 6.77 × 10^–5^, CMAC = 6), and low-frequency PTVs on *ARPC5* (*p*-value = 4.15 × 10^–5^, CMAC = 39), were associated with stroke (Fig. 3B, Fig. S5, Tables 3 and S5).

### Replication of gene aggregate findings

**Figure 4:**
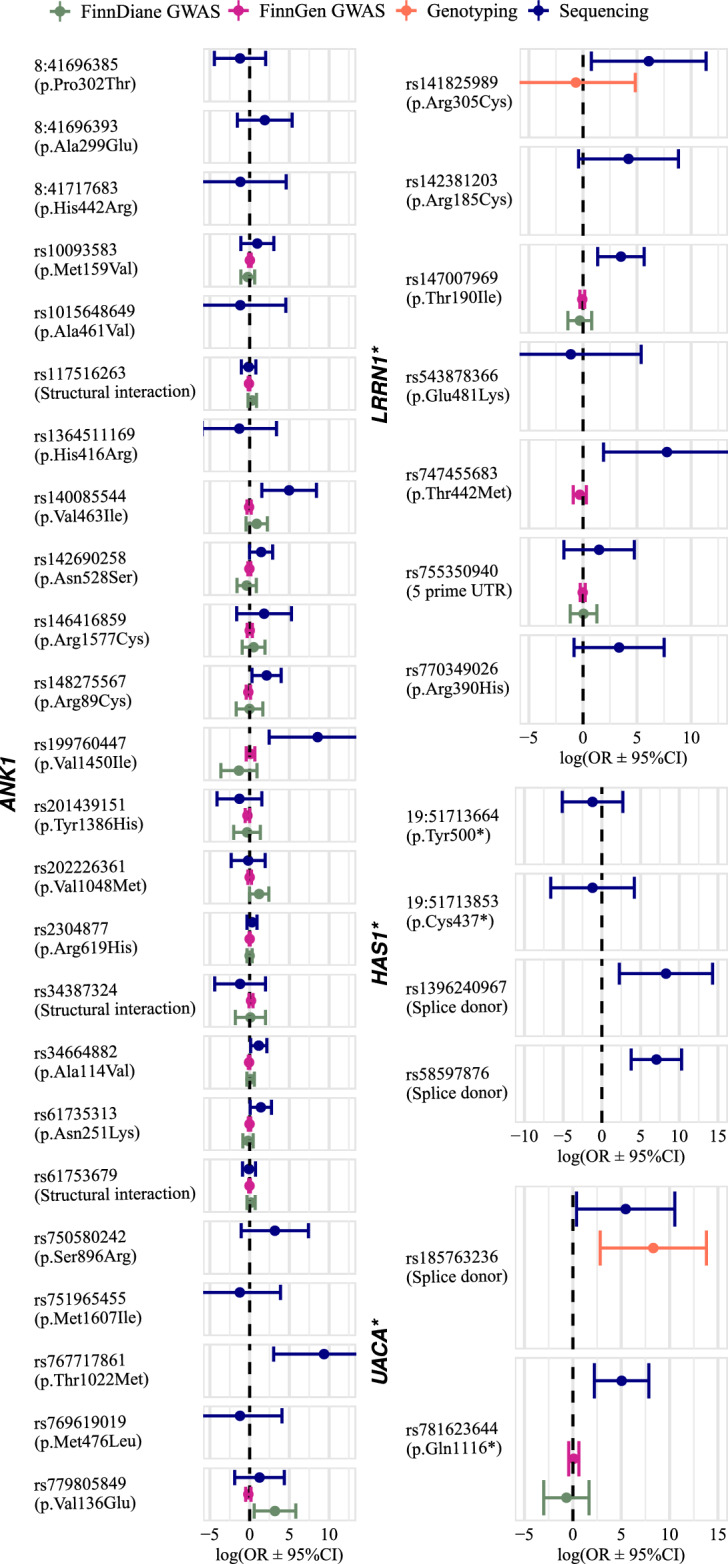
Variants in in *ANK1*, *LRRN1*, *HAS1*, and *UACA* (SKAT-O). X-axis is zoomed in, affecting many variant error bars. *Diabetic kidney disease adjusted stroke model.

We attempted T1D specific replication within the FinnDiane GWAS data, by including also five directly genotyped variants, both using the gene aggregate approach and by inspecting the exonic variants individually. Despite the uncertainty of genotype imputation and our limited statistical power for rare variants, *ANK1* and *LRRN1* showcased weak evidence of replication in T1D: Although *ANK1* did not reach significance for stroke with SKAT-O (Tables 4 and S6), one of the available fifteen variants was associated with stroke (rs779805849, *p*-value = 0.017) (Table 3, Fig. 4), and two additional variants with hemorrhagic stroke (rs146416859 and rs61753679, *p*-value < 0.05) (Table S4). *LRRN1* did not replicate for stroke in FinnDiane with rare PAVs (*p*-value = 0.50, N_variant_ = 4) (Tables 4 and S7). However, when we extended the model to low-frequency PAVs (Tables 4 and S7), thus improved statistical power and imputation quality, *LRRN1* replicated for ischemic stroke (*p*-value = 0.039, N_variant_ = 6). *UACA* contained two rare PTVs associated with stroke, of which one replicated through genotyping (*p*-value = 0.0030, Tables 3 and S5). However, the variant was ultra-rare, and replication thus uncertain. We were unable to replicate *HAS1* in T1D due to missing data; we directly genotyped one variant but found no rare allele carriers. *ARPC5* did not replicate.

**Table 4:** Lead genes with replication in individuals with T1D and in the general population.

Gene	FinnDiane adjustment	Cohort		Model		*p*-value		
		Population	Data	Variant	MAF	Stroke	Ischemic	Hemorrhagic
**Significant stroke risk genes with evidence of replication in the study**								
*ANK1*	Minimal	T1D	Discovery	PAV	< 5%	**2.23 × 10**^**–6**^	**1.31 × 10**^**–6**^	0.033
		T1D	GWAS	PAV	< 5%	0.64	0.79	0.63
		General	UKBB^27^	M3	< 1%	0.35	0.79	–
*LRRN1*	Minimal + DKD	T1D	Discovery	PAV	< 1%	**3.49 × 10**^**–6**^	8.69 × 10^–6^	0.0081
		T1D	GWAS	PAV	< 1%	0.50	0.63	0.43
		T1D	GWAS	PAV	< 5%	0.73	**0.039**	0.20
		General	UKBB^27^	M3	< 1%	0.18	0.10	–
		General	UKBB^27^	M3	< 0.001%	**0.026**	0.20	–
* HAS1*	Minimal + DKD	T1D	Discovery	PTV	< 1%	**3.11 × 10**^**–5**^	**1.17 × 10**^**–7**^	–
		General	UKBB^27^	M1	< 1%	**0.035**	0.96	–
		General	UKBB^23^	*Damaging*	< 0.1%	**0.012**	0.46	–
		General	UKBB^27^	M3	< 1%	**0.0065**	0.077	
* UACA*	Minimal + DKD	T1D	Discovery	PTV	< 1%	**6.77 × 10**^**–5**^	–	–
		T1D	GWAS	PTV	< 1%	0.42	0.23	0.83
		General	UKBB^27^	M1	< 1%	0.12	0.86	–
		General	UKBB^27^	M1	< 0.01%	**0.035**	1.00	–
**Suggestive stroke risk genes with evidence of gene aggregate replication**								
*FOXO1*	Minimal	T1D	Discovery	PAV	< 1%	1.32 × 10^–5^	1.38 × 10^–5^	–
		T1D	GWAS	PAV	< 1%	0.81	0.68	**0.017**
		General	UKBB^27^	M3	< 1%	0.055	0.57	–
		General	UKBB^27^	M3	Singleton	**0.016**	0.44	–
*TARBP2*	Minimal + DKD	T1D	Discovery	PAV	< 1%	2.06 × 10^–5^	**1.51 × 10**^**–7**^	–
		T1D	GWAS	PAV	< 1%	0.078	0.47	**1.66 × 10**^**–5**^
		General	UKBB^27^	M3	< 1%	0.44	0.90	–
*MAP3K12*	Minimal + DKD	T1D	Discovery	PAV	< 1%	1.45 × 10^–5^	**1.72 × 10**^**–7**^	0.0015
		T1D	GWAS	PAV	< 1%	0.74	0.78	0.054
		General	UKBB^27^	M3	< 1%	**0.038**	0.11	–
		General	UKBB^27^	M3	< 0.01%	0.052	**0.0058**	–
*UTS2*	Minimal	T1D	Discovery	PAV	< 5%	1.62 × 10^–5^	2.82 × 10^–5^	0.30
		T1D	GWAS	PAV	< 5%	0.24	0.35	0.29
		General	UKBB^27^	M3	< 1%	0.31	0.65	–
		General	UKBB^27^	M3	Singleton	**0.045**	0.11	–

Table represents gene aggregate replication with the best matching model to the discovery stage, and if successfully observed in further models, replication is presented. T1D GWAS (FinnDiane) may include also directly genotyped variants. UKBB (UK Biobank) replication entail WES analysis with several models, i.e., **M1**: LoF variants^27^, **M3/Damaging**: LoF and predicted-damaging missense variants^23,27^. Discovery stage *p*-values are bolded, if significant after multiple testing correction, and replication, if nominally significant (*p*-value < 0.05).

We further attempted replication in the general population by look-ups from two UK Biobank WES studies^23,27^ (Tables 4, S8 and S9). Importantly, *HAS1* replicated for stroke with rare loss-of-function variants (MAF ≤ 1%: *p*-value = 0.035^27^) and with ultra-rare deleterious variants (MAF ≤ 0.1%: *p*-value = 0.012^23^), while *UACA* replicated with ultra-rare deleterious variants (MAF ≤ 0.01%: *p*-value = 0.035^27^). Finally, *LRRN1* replicated for stroke with an ultra-rare deleterious variant model (MAF ≤ 0.001%: *p*-value = 0.026^27^), although not for ischemic stroke. *ANK1* did not replicate with the deleterious missense variant model in the general population^27^.

Out of the suggestive genes, *FOXO1*, *TARBP2*, and *MAP3K12* showcased weak replication in T1D (Tables 4, S4-S7). One variant within *FOXO1* replicated for hemorrhagic stroke with the minimal adjustment (*p*-value = 0.012), two within *MAP3K12* for hemorrhagic stroke with the additional DKD adjustment (*p*-value = 0.013); and *TARBP2* replicated for hemorrhagic stroke with the minimally adjusted SKAT-O (*p*-value = 2.59 × 10^–4^, MAF ≤ 1%). UK Biobank general population gene burden WES analysis look-ups supported stroke associations for *UTS2*, *MAP3K12*, and *FOXO1* (Tables 4 and S9)^23,27^.

### Known Mendelian stroke genes in T1D

Variants on Mendelian stroke risk genes may for instance cause small vessel disease or cerebral cavernous malformations, which can eventually lead to stroke^9^. We inspected the association of 17 autosomal genes previously linked to stroke through nonsynonymous variants (*ABCC6*, *KRIT1*, *ADA2*, *COL3A1*, *COL4A1*, *COL4A2*, *COLGALT1*, *HTRA1*, *NOTCH3*, *RNF213*, *TREX1*, *CCM2*, *PDCD10*, *CTSA*, *APP*, *CST3*, *ITM2B*)^19^ (Fig. S7). Rare PAVs on *KRIT1* were associated with stroke (*p*-value = 0.018) and ischemic stroke (*p*-value = 0.0092). Furthermore, rare PAVs on *ADA2* and on *TREX1* were associated with hemorrhagic stroke (*p*-value = 0.027 and *p*-value = 0.010, respectively). Loss-of-function variants on *KRIT1* cause vascular malformations, while *ADA2* has been linked to autoinflammatory small vessel vasculitis and *TREX1* to small vessel disease^9,19^*.*

### Sliding-window analyses

**Figure 5:**
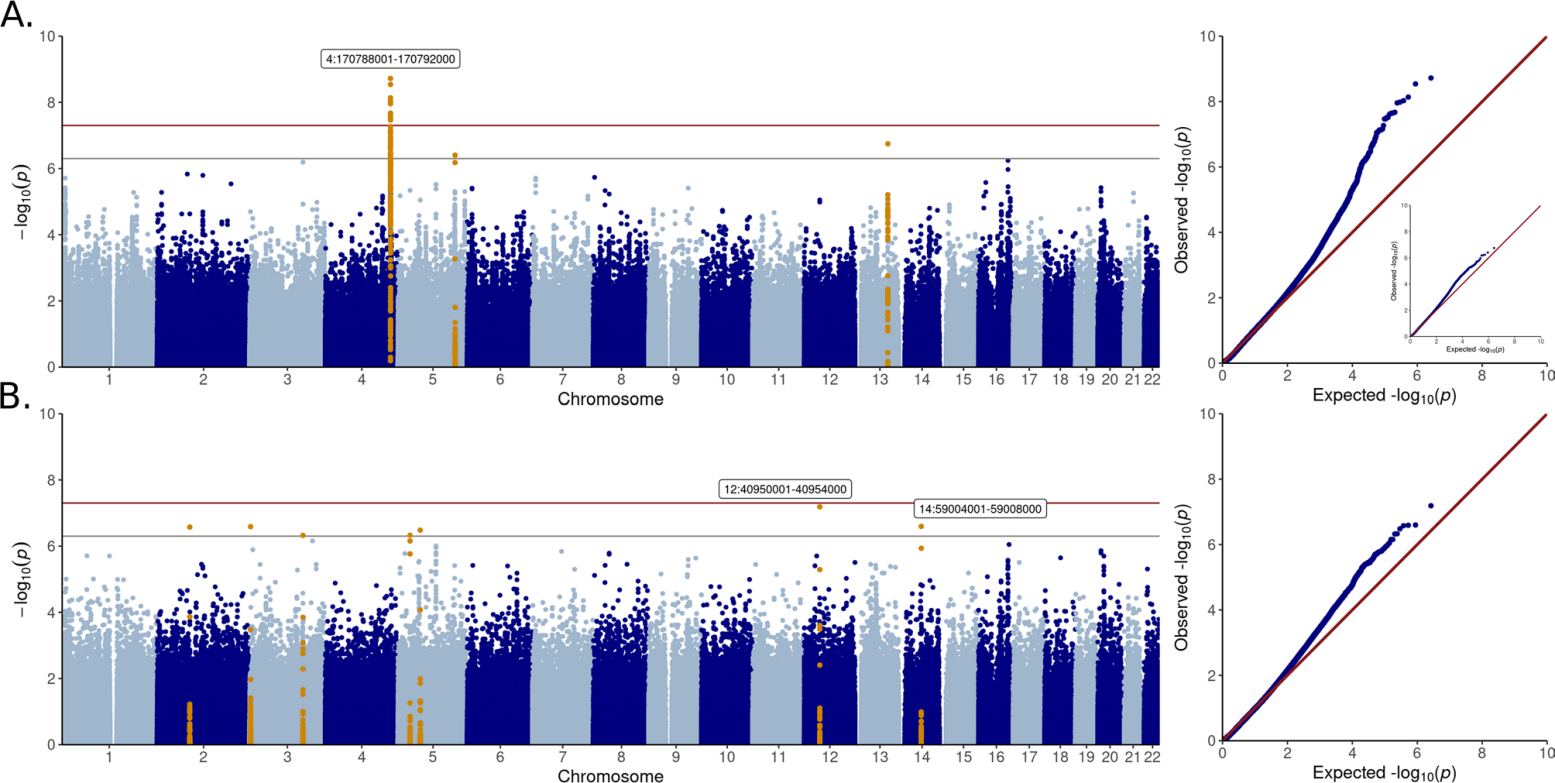
STAAR sliding-window analyses. (**A**) MAF ≤ 5% (inserted QQ-plot without the 4q33-34.1 region), and (**B**) MAF ≤ 1%.

To increase statistical power for low-frequency and rare variants on non-coding regulatory regions, we performed genome-wide sliding-window aggregate analyses with the minimal adjustment. We found further evidence for the 4q33-34.1 genomic region as we discovered fourteen windows within the region, with a genome-wide significant association between an aggregate of low-frequency variants and stroke (MAF ≤ 5%; Fig. 5A, Table S10). Importantly, two of these windows (4:170782001–170786000, *p*-value = 3.40 × 10^–8^, CMAC = 934; and 4:170784001–170788000, *p*-value = 1.10 × 10^–8^, CMAC = 1190) and ten individual variants within the 4q33-34.1 genomic region replicated for stroke in T1D (FinnDiane GWAS: *p*-value < 0.05; Table S11). To identify the most likely effector genes for the 4q33-34.1, we inspected variant expression quantitative trait loci (eQTL) from GTEx Portal and eQTLGen Consortium^28^, and functional genomics from the 3D Genome Browser^29^. 4q33-34.1 is located in the same topologically associating domain with distal promoters of *GALNTL6*, *MFAP3L* and *AADAT* in the frontal lobe and hippocampus (Fig. S8). In addition, promoter capture high-throughput chromosome conformation capture (PCHi-C) links could be identified for a few individual variants, e.g., for *GALNTL6* in the hippocampus, and *AADAT* and *MFAP3L* in the dorsolateral prefrontal cortex.

When we inspected rare variants (MAF ≤ 1%), we discovered multiple suggestive windows, e.g., close to or within the *CNTN1*, *CNTN4*, *LINC01500*, and *TGOLN2* genes (Fig. 5B, Table S10). In stroke subtype analysis, the *CNTN1* window was genome-wide significantly associated with hemorrhagic stroke (12:40950001–40954000: *p*-value = 2.10 × 10^–8^, CMAC = 24). Interestingly, *CNTN1* and *CNTN4* are located on different chromosomes, but belong to the same contactin protein family; however, replication is pending. The suggestive window near *LINC01500* (14:59004001–59008000: *p*-value = 2.53 × 10^–7^, CMAC = 19) replicated for stroke in T1D (FinnDiane GWAS: *p*-value = 0.015, CMAC = 56). Four variants within the window were available in the FinnGen general population GWAS, and one replicated for stroke (rs1281241634, *p*-value = 0.029) (Table S11). According to PCHi-C, the *LINC01500* intronic window looped to the *DACT1* promoter on the dorsolateral prefrontal cortex (Fig. S9). Finally, the *TGOLN2* window replicated for hemorrhagic stroke in T1D (FinnDiane GWAS: *p*-value = 0.037).

### Promoters and enhancers

As a more targeted aggregate approach to explore the non-coding genome, we studied rare and low-frequency variants on established regulatory regions using the minimal adjustment. We discovered three enhancers with suggestive stroke-associated enrichment of rare or low-frequency variants within intronic regions of *TRPM3*, *LOC105378983,* and *BDNF*, encoding brain-derived neurotrophic factor (Tables S12 and S13, Fig. S10). The *BDNF* enhancer was significant after multiple testing correction for ischemic stroke (*p*-value = 1.01 × 10^–6^, CMAC = 6). Regional aggregate replications were not possible in the T1D specific GWAS (N_variant_ < 2), and individual variants were missing or did not replicate. PCHi-C linked the *BDNF* enhancer to its promoter on specific brain regions (Fig. S9).

We did not identify stroke-associated promoters after correction for multiple testing (*p*-value < 3 × 10^–7^, Fig. S11). The strongest associations were two *TGOLN2* promoters (*p*-value = 5.60 × 10^–6^, CMAC = 9, MAF ≤ 1%), located on the previously mentioned *TGOLN2* window, and a *TRPM2-AS* promoter (*p*-value = 5.78 × 10^–6^, CMAC = 33, MAF ≤ 1%; Tables S14 and S15). The aggregate of rare variants on *TRPM2-AS* promoter nearly replicated for stroke in T1D (FinnDiane GWAS: *p*-value = 0.053). When we inspected variants individually, one out of nine available variants replicated in the general population for ischemic stroke (FinnGen GWAS:* p*-value = 0.038). In GTEx, rs762428 within the *TRPM2-AS* promoter associated significantly to *TRPM2* level in whole blood (NES = -0.63) and lungs (NES = -0.41, *p* < 0.001), also nominally in other tissues such as the hypothalamus (NES = -0.42). *TRPM2* encodes a calcium-permeable and non-selective cation channel expressed mainly in the brain. The gene has been linked to ischemic stroke^30^, and belongs to the same protein subfamily as the above mentioned *TRPM3*. *TRPM2* inhibitors have been proposed as a drug target for central nervous system diseases^31^, thus, our results suggested that these inhibitors could be beneficial also for stroke in T1D, although further validation of the genetic associations are needed.

**Figure 6:**
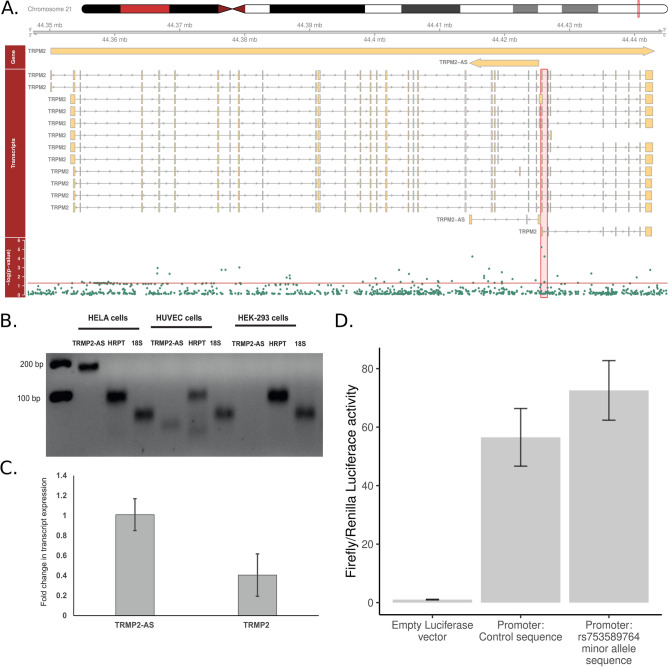
*TRPM2-AS* regional plot and experimental data. (**A**) Regional plot of *TRPM2* and *TRPM2-AS* extended region (Gviz R package 1.38.3^61^); the discovered promoter is highlighted (red). (**B**) Semi-quantitative RT-PCR detecting *TRPM2-AS* transcript in HELA cells, but not in HUVEC and HEK-293 cells (hypoxanthine phosphoribosyltransferase 1 (*HPRT1*) and 18S ribosomal RNA as positive control), (**C**) Relative expression of *TRPM2-AS* and *TRPM2* transcripts in HELA cells (*HPRT1* used as reference transcript to normalize quantitative RT-PCR), (**D**) Firefly/Renilla luciferase assay of promoter activity. Empty vector (mean = 1.0, 12 technical repeats) as transfection control for baseline luciferase activity was compared to *TRPM2-AS* control promoter, i.e., major allele in all identified *TRPM2-AS* variants (mean = 56.5, 11 technical repeats, *p*-value = 0.00022); which was further compared to a *TRPM2-AS* promoter with rs753589764 minor allele (mean = 72.6, 11 technical repeats, *p*-value = 0.27): All cloned before firefly reporter gene to evaluate potential transcriptional promoter activity. Statistical significance was assessed with Student’s t-test and error bars represent standard error.

We performed luciferase promoter analysis of the stroke-associated sequence within the *TRPM2-AS* promoter region to experimentally confirm its promoter activity (Fig. 6). As we detected *TRPM2-AS* expression in HELA cells but not in HUVEC or HEK-293 cells using semi-quantitative RT-PCR, the luciferase analysis was performed in HELA cells, which indicated strong promoter activity. The most strongly stroke-associated variant, rs753589764, did not significantly affect luciferase activity under normal cell culture conditions (*p*-value = 0.27, 22 technical repeats). However, we cannot rule out a variant effect under cellular stress, e.g., oxidative stress, or in other cell lines, and therefore, further promoter experiments should be performed in future.

## Discussion

Stroke heritability has been estimated to range between 30 and 40%, but the genomic loci identified thus far explain only a small fraction of heritability^9^. One potential explanation underlying the missing heritability are rare variants missed by GWAS. Therefore, we performed WES and WGS in a total of 1,051 Finnish individuals with T1D to discover rare and low-frequency variants associated with stroke and its major subtypes, either specific for T1D, or generalizable to the non-diabetic population. We identified multiple significant loci with evidence of replication, including protein altering or truncating variants on *ANK1*, *HAS1*, *UACA*, and *LRRN1*, as well as a 4q33-34.1 intergenic region.

With single variant analyses, we identified a missense variant on *SREBF1* (rs114001633, p.Pro227Leu), which was exome-wide significantly associated with stroke, and further replicated for hemorrhagic stroke in T1D. As the variant was ultra-rare, and we had a relatively small number of hemorrhagic stroke cases, further replication is needed in T1D to conform this finding. *SREBF1* encodes a transcription factor involved in lipid metabolism and insulin signaling^32^.

Gene aggregate tests (SKAT-O) detected four genes within which PAVs (*ANK1* and *LRRN1*) or PTVs (*HAS1* and *UACA*) were associated with stroke with evidence of replication; *LRRN1*, *HAS1*, and *UACA* after adjustment for DKD. *ANK1* did not replicate in T1D with the gene aggregate approach, however, one out of the fifteen available variants replicated for stroke in T1D (rs779805849, p.Val136Glu). Of note, SIFT and PolyPhen predicted many *ANK1* variants as deleterious^33,34^. *ANK1* encodes ankyrin-1, within which variants cause hereditary spherocytosis, an inherited disease that changes the shape of red blood cells^35^. Previous genome-wide association studies have linked the gene to T2D^36^, while another gene from the ankyrin protein family, *ANK2*, is a previously identified stroke risk locus^37^.

Rare PAVs on *LRRN1* were associated with stroke. *LRRN1* did not replicate with the corresponding model in T1D, however; with a model extended to low-frequency PAVs, *LRRN1* replicated for ischemic stroke. Rare variant replication is problematic with GWAS data due to the uncertainty of the imputation, which may explain the need of increasing the allele frequency threshold to observe a successful replication. Furthermore, *LRRN1* was nominally associated with stroke in the general population through an aggregate of ultra-rare loss-of-funtion and deleterious missense variants^27^. *LRRN1* encodes leucine rich repeat neuronal protein 1, with a brain-enriched expression profile.

*HAS1* consistently replicated for stroke with rare loss-of-funtion and deleterious variant aggregate models in the general population^23,27^, while *UACA* replicated for stoke with one ultra-rare deleterious variant model^27^. *HAS1* encodes an enzyme producing hyaluronan and with expression induced by inflammation and glycemic stress^38^. Of note, an increased hyaluronan turnover has been suggested to follow ischemic stroke^39^. No additional *HAS1* PTV carriers were identified among the T1D replication cohort, thus, a diabetes-specific replication is pending. Nevertheless, *HAS1* PTVs may be of particular importance in T1D, as dysregulation of endothelial glycocalyx hyaluronan has been suggested to contribute to diabetic complications^40^. Finally, it must be noted that PTVs have not been functionally confirmed as loss-of-function, but the annotations are predictions; PTV at the beginning of a gene is likely more severe than at the end, and in fact, PTVs closer to the *HAS1* transcription start site were more strongly associated with stroke.

To increase statistical power on regulatory regions, we performed statistical aggregate tests in genomic windows, enhancers and promoters^41,42^. Of note, we extended genomic window length from the default to increase statistical power, which however also reduced precision as the causal region might be narrower. We found fourteen genome-wide significant stroke-associated windows with low-frequency variants on 4q33-34.1, of which two replicated for stroke in T1D. According to eQTLs and PCHi-C interactions, 4q33-34.1 variants most likely target *GALNTL6*, *MFAP3L* or *AADAT*. We also discovered a suggestively stroke-associated window through rare variants within *LINC01500*, which replicated for stroke in T1D. According to PCHi-C, the *LINC01500* window targets a promoter of *DACT1*. Finally, an aggregate of rare variants was suggestively associated with stroke on *TRPM2-AS* promoter, which nearly replicated in T1D (*p*-value = 0.053). Importantly, transient receptor melastatin 2 (*TRPM2*) has been previously associated with ischemic stroke^30,31^. Our functional cell-based assay validated the *TRPM2-AS* region promoter activity. However, the most strongly stroke-associated variant, rs753589764, did not associate with *TRPM2-AS* promoter activity under normal cell culture conditions in HELA cells.

Limitations of the study include the limited statistical power due to moderate sample size at the discovery stage, replication of rare variants with imputed GWAS data, and non-conservative statistical estimates for the rarest variants due to case–control imbalance (≈1:6), especially for the stroke subtypes. We were able to improve the statistical power on exomes by meta-analyzing WES and WGS, and we performed the stroke-subtype specific analyses only for a limited number of suggestive findings to avoid spurious signals due to unstable statistical estimates. To further improve statistical power, we performed statistical aggregate tests on gene exons and on intergenic regions, i.e., enhancers, promoters, and genomic windows. Of note, we studied only transcribed enhancers, and thus, some enhancers could have been missed. We defined promoters with an arbitrarily selected 1,000 bp extension downstream TSS, which may not have always been optimal as the promoter lengths vary. Further limitations are the lack of sequencing-based replication data in individuals with T1D, and that we regarded nominal significance as replication (*p*-value < 0.05). However, we sought for replication by combining available data sources, i.e., FinnGen (Finnish general population GWAS), UK Biobank (general population WES), and FinnDiane (GWAS and genotyping in Finnish individuals with T1D). Of note, stroke cases were younger and had a shorter diabetes duration than controls in the FinnDiane cohorts; the difference being the most extreme in the discovery cohorts, which may have imposed unsuccessful replication for variants with an age or diabetes duration dependent effect. Importantly, gene burden variant selection criteria did not perfectly match to ours within UK Biobank WES^23,27^, especially with the low-frequency protein altering variant models, which may explain some unsuccessful gene aggregate replications. Finally, while conducting the analyses in an isolated population has certain advantages for variant discovery, it also raises the question of generalizability of the findings to other populations. In addition to the replication attempted in the UK Biobank, further research is needed to validate our findings in non-Finnish individuals with T1D.

The strengths of this study include a well characterized cohort and comprehensively performed single variant and aggregate analyses both for the coding and non-coding regions of the genome. Stroke is a challenging phenotype to address with ICD codes and many loci associated with rare stroke phenotypes may go unnoticed even with large population-wide genetic studies. We performed analyses for well-defined stroke phenotypes verified by trained neurologists. Furthermore, as we conducted the analyses in specific high-risk individuals from an isolated population, thus with less genetic and phenotypic diversity, we had improved statistical opportunities to identify genetic risk loci.

In conclusion, we studied rare and low-frequency stroke-associated genetic variants with whole-exome or whole-genome sequencing in 1,051 individuals with T1D and report the first genome-wide study on stroke genetics in diabetes. The results highlight 4q33-34.1, *SREBF1*, and *ANK1* for stroke in T1D; and *HAS1*, *UACA*, *LRRN1*, *LINC01500*, and *TRPM2-AS* promoter as stroke risk loci that likely generalize to the non-diabetic population. The represented results require future validation with next-generation sequencing in a larger cohort of individuals with T1D.

## Methods

### Materials

We studied WGS in 571 and WES in 480 non-related individuals with T1D, entailing 112 and 74 stroke cases, respectively (Table 1, Table S1, Fig. S1 and S2). Patients in WGS and WES were non-overlapping. The patient selection for both data sets were originally designed for DKD, such that half of the individuals had severe DKD, and half had no DKD (i.e., normal albumin excretion rate) despite a long duration of T1D^21,43^. Importantly, this resulted in stroke cases being younger and having shorter diabetes duration than controls, contradictory to presumption. Individuals in the present study were diagnosed with T1D by their attending physician and had diabetes onset age < 40 and insulin initiated within one calendar year from the diabetes diagnosis. Stroke cases were identified for the participants from Finnish registries based on ICD codes until the end of 2017 (Table S16). The phenotypes were verified, and stroke cases classified into ischemic- and hemorrhagic strokes by trained neurologists using medical files and brain imaging data. For individuals without data verified by neurologists available (N_WGS_ = 27, N_WES_ = 2), we considered only the registry data, excluded controls with intermediate stroke phenotypes (e.g., transient ischemic attack), and were unable to classify stroke cases into ischemic- and hemorrhagic subtype. Importantly, we required stroke to have occurred after T1D diagnosis, and controls to have > 35 years of age and > 20 years of diabetes duration. Next-generation sequencing data was processed to GRCh38 reference panel, and variants annotated with SNPEff v.5 software^44^ (Fig. S12). In variant QC, for autosomal variants, we required Hardy–Weinberg equilibrium (HWE) *p*-value > 10^–10^ and variant call rate > 98%; and for X chromosome variants, only variant call rate > 98%. The pipeline is described in Detailed Methods of the Supplementary Information.

Within the FinnDiane study, we have GWAS data for almost the entire cohort, i.e., 6,458 individuals with T1D or their relatives. GWAS data has been previously processed to GRCh37 reference genome. However, we have now lifted the genotyping positions over to GRCh38, re-imputed the data to SISu v3 reference panel, and annotated with SNPEff v.5 software^44^ (Fig. S13). We attempted replication in individuals with T1D within the FinnDiane GWAS data, non-overlapping to sequencing data (N = 3,945, Table S17 and S18, Fig. S14), and restricted to high imputation quality variants (*r*^2^ > 0.80), and by directly genotyping twelve lead variants for replication (N = 3,263, Table S19, Fig. S15). Stroke cases were younger and had shorter diabetes duration than controls in the replication cohorts, comparably to the discovery cohorts, although with a less extreme difference. Of note, variant genotyping was performed with one Agena iPlex multiplexing assay at the Institute for Molecular Medicine Finland, Helsinki, Finland (Table S20), and the genotyping replication limited to individuals within GWAS data in order to perform relatedness adjustment. Stroke phenotype and control criteria within replication in T1D were defined similarly to the WES and WGS data.

### Single variant analyses

We analyzed the genome with an additive inheritance model. For variants available in WES and WGS data, we performed score test with rvtests (version 20190205)^45^, followed by fixed-effect inverse variance based meta-analysis (Total MAC ≥ 5, and MAC ≥ 2 in WES and WGS) with metal (version 20110325)^46^. For variants available only in one data set we utilized exact Firth regression (MAC ≥ 5)^45^. Importantly, Firth logistic regression has been suggested the most conservative statistical test for joint rare variant analyses, especially with case–control imbalance, while score test to have the highest statistical power for rare variant meta-analyses^47^. The additive single variant analyses were adjusted for the calendar year of diabetes onset, sex, and two first genomic data principal components (i.e., minimal adjustment setting), and additionally for DKD, which is one of the most important risk factors of stroke in T1D^48^. WGS and WES stroke controls are older and have longer T1D duration than cases—contrary to true stroke predisposition—due to next-generation sequencing patient selection optimization for DKD by considering T1D duration. Thus, in order to avoid statistical bias, we adjusted for the calendar year of diabetes onset; a major stroke risk factor correlated with age, T1D duration, and T1D treatment quality.

### Gene aggregate analyses

In order to improve statistical power for rare (MAF ≤ 1%) and low-frequency (MAF ≤ 5%) variants, we performed gene aggregate analyses with an optimal unified sequence kernel association test (SKAT-O) meta-analysis with MetaSKAT (version 0.81)^49^, separately within two distinct classes (Table S21): protein-altering variants and protein-truncating variants i.e., the more severe putative loss-of-function variants^50^. Importantly, the protein-altering variant class entail protein-truncating variants in addition to variants that alter the amino acid sequence. Of note, SKAT-O maximizes statistical power by optimally combining sequence kernel association test and burden test^51^. All variable sites (MAC ≥ 1) were accepted into gene aggregate analysis, and the aggregate tests were required to entail at least two variants (N_variant_ ≥ 2), with a cumulative MAC (CMAC) across all included variants within the gene ≥ 5. We adjusted the analyses for the calendar year of diabetes onset, sex, and the two first genomic data principal components, and additionally for DKD. We did not report genes with all variants in perfect LD, and inspected individual variant stroke-associations within the genes using the score test fixed-effects meta-analysis^45,46^. Multiple testing correction, based on the number of tested genes, resulted in significance thresholds of *p*-value < 4 × 10^–6^ for PAVs (MAF ≤ 1%: N_gene_ = 11,954; MAF ≤ 5%: N_gene_ = 13,069), *p*-value < 8 × 10^–5^ for PTVs with MAF ≤ 1% (N_gene_ = 663), and *p*-value < 6 × 10^–5^ for PTVs with MAF ≤ 5% (N_gene_ = 908). In addition, we investigated stroke-associations for 17 autosomal Mendelian stroke risk genes regardless of CMAC^19^, and were able to report associations for 13 of them.

### Sliding-window and regulatory region aggregate analyses with whole-genome sequencing

To increase statistical power for low-frequency and rare variants on intergenic regions, we performed functionally informed sliding-window analyses, i.e., aggregate analyses within 4,000 base pair (bp) regions (N_variant_ ≥ 2, CMAC ≥ 5)—separated by 2,000 bps—with variants statistically weighted according to their rarity and functional importance using STAAR-O (STAAR R package 0.9.6)^41,52^. Functional importance was defined with Combined Annotation-Dependent Depletion (CADD) data^52^ using variant MAF (to up-weight rarer variants), pre-computed CADD score, and the first annotation principal component from seven annotation classes (Fig. S16, Table S22), calculated following the guidelines^41^. Of note, the scores were utilized on the PHRED scale. We adjusted the analyses for the calendar year of diabetes onset, sex, and the two first genomic data principal components.

We studied established regulatory regions, i.e., enhancers and promoters (N_variant_ ≥ 2, CMAC ≥ 5), as defined in FANTOM5 cap analysis of gene expression (CAGE) human data reprocessed to the GRCh38 reference genome^42^, with promoters defined as the transcription start site (TSS) extended to 1,000 bp, and weighted by the variant rarity in PHRED scale. FANTOM5 atlases have been measured with multiple human primary cell lines, tissues, and cancer cell lines^53,54^. The regulatory regions were analyzed with STAAR R package 0.9.6^41^, by adjusting for calendar year of diabetes onset, sex, and two first genomic data principal components. With low-frequency variants, the multiple testing corrected significance thresholds were *p*-value < 2.9 × 10^–7^ for promoters (N_region_ = 172,134) and *p*-value < 2.6 × 10^–6^ for enhancers (N_region_ = 19,472). For rare variants, the thresholds were *p*-value < 3.5 × 10^–7^ (N_region_ = 141,779) and *p*-value < 4.3 × 10^–6^ (N_region_ = 11,665), respectively. We did not report regions with all variants in perfect LD.

### Replication

Within the FinnDiane GWAS data, we attempted replication of high imputation quality genetic variants (*r*^2^ > 0.80) with score test (rvtests 20190205^45^) and had good statistical power (> 80%) to detect a nominal association with an odds ratio (OR) ≥ 2.5 for additive low-frequency variants (MAF = 1%) (Fig. S17)^55^. However, for rare variants with MAF = 0.1% and OR < 9, we had only limited power to detect an association even with nominal significance (*p*-value < 0.05). Thus, we considered nominal significance as the replication threshold (*p*-value < 0.05). We attempted direct genotyping for replication for twelve variants, but minor allele carriers were observed only for seven of them (Table S20). We performed single variant analyses for the genotyped variants similarly with score test, except for one *LRRN1* variant with linear regression and no relatedness adjustment (stats R package 4.2.1) due to lack of alternative allele carriers among individuals with the required relatedness information. Most variants within the aggregate discoveries were rare or ultra-rare (MAF≈0.1%), making replication with imputed genomic data problematic. Nevertheless, we attempted replication within the FinnDiane GWAS data (*r*^2^ > 0.80) by including also the directly genotyped variants (SKAT-O, STAAR-O). We performed SKAT-O using GMMAT R package 1.3.2 by imputing missing genotype dosages to mean^56^, while intergenic aggregate analyses were performed similarly with STAAR R package^41^. Replication analyses were adjusted comparably to the discovery stage analyses, except that relatedness in replication was accounted for with relatedness matrices instead of genomic principal components (Balding-Nichol’s approximation kinship matrix in single variant analysis and GEMMA relatedness matrix in aggregate analyses)^45,57^. We attempted replication in the general population for genetic variants from the large-scale population-wide FinnGen project release 6 GWAS data with phenotypes best matching our definitions (https://www.finngen.fi/en) (Table S23), and for the gene aggregate discoveries from UK Biobank summary statistics^23,27^. Of note, no proxies in LD were found for the lead single variant findings (rs4435704, rs4401420), and thus, we did not consider linkage disequilibrium in replication beyond the traditional imputation approach.

### Functional characterization of the genetic variants and regions

We inspected genetic variant characteristics from GTEx Portal, eQTLGen Consortium (*p*-value < 0.05)^28^, RegulomeDB^58^, YUE Lab^29^, and the Ensemble Variant Effect Predictor^33,34,59^. Functional characterization of the *TRPM2-AS* promoter is described in Detailed Methods of the Supplementary Information. In short, we assessed *TRPM2-AS* expression in three cell lines (HELA, HEK-293, HUVEC) and noted expression in HELA cells. We then assessed the influence of the chromosomal location and the genotype of the most strongly stroke-associated variant (rs753589764) on promoter activity in HELA cells under normal cell culture conditions with a dual-luciferase reporter assay (22 technical repeats).

### Detailed methods

Detailed Methods are available in the Supplementary Information.

### Ethical approval

The study protocol has been approved by the ethics committee of the Helsinki and Uusimaa Hospital District (491/E5/2006, 238/13/03/00/2015, and HUS-3313-2018), and performed in accordance with the Declaration of Helsinki. All participants gave informed consent before participation.

### Supplementary Information

Supplementary Information 1.

Supplementary Information 2.

Supplementary Information 3.

## Data Availability

Individual-level data for the study participants are not publicly available, because of the restrictions due to the study consent provided by the participant at the time of data collection. The readers may propose collaboration to research the individual level data with correspondence with the lead investigator. Gene aggregate test stroke summary statistics are provided in the Supplementary Data.

## Code availability

We utilized public softwares for the statistical analyses: plink2 (https://www.cog-genomics.org/plink/2.0/), metal (http://csg.sph.umich.edu/abecasis/metal/), rvtests (http://zhanxw.github.io/rvtests/), metaSKAT (https://cran.r-project.org/web/packages/MetaSKAT/), STAAR-O (https://github.com/xihaoli/STAAR), and GMMAT (https://github.com/hanchenphd/GMMAT).
