# Supplementary material for: Whole-genome sequencing identifies variants in ANK1, LRRN1, HAS1, and other genes and regulatory regions for stroke in type 1 diabetes
Source: Sci Rep. 2024 Jun 11;14:13453. doi: 10.1038/s41598-024-61840-7 (PMC11166668; doi:10.1038/s41598-024-61840-7)
Supplement: Supplementary file 2 — Supplementary Information 2. [file 41598_2024_61840_MOESM2_ESM.pdf]

Whole-genome sequencing identifies variants in *ANK1*, *LRRN1*, *HAS1,* and other genes and regulatory regions for stroke in type 1 diabetes

Anni A. Antikainen, Jani K. Haukka, Anmol Kumar, Anna Syreeni, Stefanie Hägg-Holmberg, Anni Ylinen, Elina Kilpeläinen, Anastasia Kytölä, Aarno Palotie, Jukka Putaala, Lena M. Thorn, Valma Harjutsalo, Per-Henrik Groop and Niina Sandholm, on behalf of the FinnDiane Study Group

Supplementary Material

Detailed Methods 1
Participants and stroke definitions 1
Sequencing material 1
Replication material 2
Statistical methods 2
Single variant analyses 2
Gene aggregate analyses 3
Sliding-window analyses and regulatory regions 3
Regional plots and functional characterization 4
Functional research on *TRPM2-AS* promoter 4
Single variant replication 4
Gene aggregate replication 4
Sliding-window and regulatory region replication 4
Supplementary Figures 5
Fig. S1: Clinical characteristics of individuals in WGS 5
Fig. S2: Clinical characteristics of individuals in WES 6
Fig. S3: Single variant analysis with DKD adjustment 7
Fig. S4: SKAT-O minimal model Manhattan plot 8
Fig. S5: SKAT-O with additional DKD adjustment Manhattan plot 9
Fig. S6: *MAP3K12* and *TARBP2* regional plot 16
Fig. S7: Known Mendelian stroke-risk genes in T1D 17
Fig. S8: Topologically associating domain (TAD) on 4q33-34.1[24] 18
Fig. S9: Enhancer variant PCHi-C links[24] 19
Fig. S10: Stroke enhancer association Manhattan plot 20
Fig. S11: Stroke promoter association Manhattan plot 21
Fig. S12: Sequencing data processing pipeline (WES and WGS) 22
Fig. S13: GWAS replication data processing pipeline (FinnDiane) 23
Fig. S14: Clinical characteristics of individuals in FinnDiane GWAS 24
Fig. S15: Clinical characteristics of individuals in genotyping 25

Fig. S16: Annotation PCA within functional classes (CADD) ... 26
Fig. S17: Statistical power in replication ... 27
Fig. S18: PCA of sequencing data (WES and WGS) ... 28
Fig. S19: *TRPM2-AS* expression in HELA, HEK-293 and HUVEC cell lines ... 29
Supplementary Tables ... 30
Table S1: Stroke subtype sequencing data clinical characteristics ... 30
Table S16: Stroke ICD codes in Finnish registry data ... 31
Table S17: GWAS replication clinical characteristics (FinnDiane) ... 32
Table S18: GWAS stroke subtype replication clinical characteristics ... 33
Table S19: FinnDiane genotyping clinical characteristics ... 34
Table S20: Genotyped variants in FinnDiane ... 35
Table S21: Variant type classification for SKAT-O ... 36
Table S22: CADD functional annotations within annotation classes ... 37
Table S23: FinnGen replication ICD codes (GWAS) ... 38
Table S24: Physicians and nurses at health care centers participating in the collection of FinnDiane patients ... 39
References ... 41

Detailed Methods

Participants and stroke definitions

The study is part of the FinnDiane Study established to investigate complications of T1D. We have 490 and 583 non-related individuals with T1D with quality-controlled WES and WGS, respectively. Patient selection for WES and WGS were originally designed for DKD. For WES (N=500), the selected 250 DKD cases had rapid onset of kidney disease after T1D diagnosis (≤25 years), while the 250 DKD controls had normal albumin excretion rate despite a long T1D duration (≥32 years) and were enriched for poor glycemic control (HbA1c≥6.5%)[1]. Correspondingly, for WGS (N=600), the selected 300 DKD cases presented with severe kidney disease, and the 300 DKD controls with normal albumin excretion rate despite a long duration of T1D (≥35 years). Individuals in the present study were diagnosed with T1D by their attending physician and had diabetes onset age <40 and insulin initiated within one calendar year from the diabetes diagnosis. Stroke phenotypes were identified from the Finnish Death Registry, Statistics Finland, and the Care Register for Health Care, Finnish Institute for Health and Welfare until the end of 2017 and were verified by trained neurologists from medical files and brain imaging data. We required acute stroke events to have occurred after the T1D diagnosis. For individuals without data verified by neurologists available (N_{WGS}=27, N_{WES}=2), we considered only the registry data, and excluded five controls with transient ischemic attacks or other intermediate stroke phenotypes due to potential for misclassification (**Table S16**). Furthermore, we classified stroke events into ischemic- and hemorrhagic strokes whenever stroke subtypes verified by neurologists were available. Ischemic strokes entailed lacunar-, non-lacunar-, and unclear infarctions, while hemorrhagic strokes comprised intracerebral and subarachnoid hemorrhages. We limited controls to individuals with >35 years of age and >20 years of diabetes duration, because only ~5% of cases had experienced their first stroke event before these limits in sequencing data. Thus, we studied WES and WGS data of 480 and 571 individuals with T1D, respectively (**Table 1, Table S1, Fig. S1** and **S2**).

Sequencing material

In total, 599 individuals were succesfully whole genome sequenced at Macrogen Inc. using the Illumina HiSeq X platform (Macrogen Inc., Rockville, MD, USA) with at least 30× average coverage (1-8 lanes). Furthermore, 500 individuals with T1D were whole-exome sequenced at the University of Oxford, UK, as described earlier[1]. In short, libraries were multiplexed and captured with Illumina TruSeq™ Exome Enrichment Kit and sequenced on an Illumina HiSeq2000 with 100 bp paired end reads (1-2 lanes). Five WES samples did not pass our initial quality control (QC). For this work, the pre-processed aligned reads of 495 individuals were converted back to unaligned FASTQ-reads and re-processed with comparable pipeline to WGS.

First, we trimmed the WES and WGS sequencing reads[2]. We processed 495 WES and 599 WGS samples further according to Broad Institute's best practices guidelines with Genome Analysis Toolkit 4 (GATK4)[3] (**Fig. S12**): We aligned reads by lane to GRCh38 reference genome with Burrows-Wheeler Aligner, sorted and marked duplicate reads, recalibrated bases by chromosome, and called variants by sample. WES and WGS

were joint called separately. We filtered variants according to excess heterozygosity threshold of 54.69, truth sensitivity level 99.7%, and GATK's recommended tranche thresholds for SNPs and indels. We assessed sample concordance with FinnDiane GWAS data whenever possible. Finally, 490 and 583 individuals passed QC within WES and WGS, respectively. In variant QC, for autosomal variants, we required Hardy-Weinberg equilibrium (HWE) p-value $>10^{-10}$ and variant call rate >98%; and for X chromosome variants, only variant call rate >98%. We annotated variants with SNPEff v.5 software[4], and performed principal component analysis (PCA) with plink (v1.90b3.26)[5] to enable population stratification adjustment with genomic data principal components (**Fig. S18**). The two first genomic principal components explained 0.69% and 0.63% of variance within WES and WGS, respectively. Ancestry clusters were not detectable with the principal components (**Fig. S18**); thus, we adjusted analyses only for the two first genomic data principal components. WES and WGS comprised 324,817 and 21.92 million variants, respectively.

Replication material

In FinnDiane, we have GWAS data for 6,458 Finnish individuals with T1D and their relatives. Genomes were genotyped at the University of Virginia, and previously processed to GRCh37 reference genome by variant calling with zCall algorithm[6,7]. We lifted the genotyping positions over to GRCh38, re-imputed the data to SISu v3 reference panel, and annotated with SNPEff v.5 software[4] (**Fig. S13**). We restricted the data to 15,026,805 high imputation quality variants (r^2>0.80), and individuals to those with T1D; age at onset <40 years and insulin treatment initiated within two years from diagnosis, if insulin initiation year known, and to those not in the sequencing data. Controls were required to have diabetes duration >20 years and age >35 years. Thus, GWAS replication entailed 367 cases and 3,578 controls (**Fig. S14**, **Tables S17** and **S18**). Instead of principal component adjustment, we utilized rvtests kinship matrix with Balding-Nichol's approximation in single variant analysis and GEMMA relatedness matrix in aggregate analyses[8,9].

Twelve lead variants were selected for replication by variant genotyping in 3,600 FinnDiane participants with T1D on one Agena iPlex multiplexing assay at the Institute for Molecular Medicine Finland, Helsinki, Finland (**Table S20**). Variants were prioritized onto to the same multiplexing design based on statistical significance, and one heterozygous variant carrier within sequencing data included as the positive control. Replication was limited to individuals within GWAS data to perform relatedness adjustment with kinship matrix (N=3,263, **Table S19, Fig. S15**).

Statistical methods

Single variant analyses

We analyzed variants available in WES and WGS data with score test fixed-effect inverse variance based meta-analysis (CMAC≥5, WES and WGS: MAC≥2), and variants only available in one of the data sets with Firth regression (MAC≥5). Analyses were carried out with rvtests (version 20190205)[8] and metal (version 20110325)[10].

Gene aggregate analyses

We performed autosomal gene aggregate tests with SKAT-O, combining burden and variance-component tests, with the aim to increase statistical power and stability[11]. We performed SKAT-O meta-analysis, separately with PAVs and PTVs, between WES and WGS using MetaSKAT (version 0.81)[12], which exploits single variant score statistics. PTVs were here defined as predicted loss-of-function variants (**Table S21**). PAV analyses entail, in addition to the PTVs, variants that alter the amino acid sequence. All variable sites (MAC≥1) were accepted into gene aggregate ($N_{variant}$≥2, CMAC≥5). We did not report genes with all variants in perfect LD, and inspected individual variant stroke-associations within the genes using score test fixed-effects meta-analysis[8,10]. In summary, we performed SKAT-O analyses for 908 genes with low-frequency PTVs, 663 genes with rare PTVs, 13069 genes with low-frequency PAVs, and 11954 genes with rare PAVs. Thus, multiple testing correction based on the number of included genes resulted in significance thresholds: p-value<4×10^{-6} for PAVs (MAF≤1% and MAF≤5%), p-value<8×10^{-5} for PTVs with MAF≤1%, and p-value<6×10^{-5} for PTVs with MAF≤5%. For the known Mendelian stroke risk genes (*ABCC6*, *KRIT1*, *ADA2*, *COL3A1*, *COL4A1*, *COL4A2*, *COLGALT1*, *HTRA1*, *NOTCH3*, *RNF213*, *TREX1*, *CCM2*, *PDCD10*, *CTSA*, *APP*, *CST3*, *ITM2B*)[13], we reported gene-stroke associations whenever rare or low-frequency variant carriers were successfully observed, regardless of CMAC.

Sliding-window analyses and regulatory regions

We performed functional annotation weighted sliding window analyses on the WGS data with the STAAR R package 0.9.6[14]. For variant annotations we utilized CADD v1.6 GRCh38 data[15,16], more specifically; variant MAF (to up-weight rarer variants), pre-computed CADD score, and the first annotation principal components from seven annotation classes (**Fig. S16, Table S22**). Following guidelines[14], missing annotations were imputed to default and distributions were standardized before principal component analysis. Annotations were transformed to PHRED scale with appropriate direction. We utilized 4,000 bp windows ($N_{variant}$≥2, CMAC≥5) separated by 2,000 bp skips. We studied 20.7 million autosomal variants with available functional annotations.

As enhancers and promoters, we considered FANTOM5 CAGE profiles reprocessed to the GRCh38 reference genome (https://fantom.gsc.riken.jp/5/)[17–19]; however, we extended CAGE TSSs to form full-length promoters (1,000 bp). We analyzed regulatory regions ($N_{variant}$≥2, CMAC≥5) with the STAAR R package[14], using only allele frequencies as variant annotations, which allowed us to include more autosomal variants. With low-frequency variants, 172,134 promoters and 19,472 enhancers were analyzed, resulting in multiple testing corrected significance thresholds p-value<2.9×10^{-7} and p-value<2.6×10^{-6}, respectively. For rare variants, the thresholds were p-value<3.5×10^{-7} and p-value<4.3×10^{-6}, respectively. We did not report regions with all variants in perfect LD.

Regional plots and functional characterization

We produced regional association plots with LocusZoom[20] and Gviz R package 1.38.3[21]; and inspected variant characteristics from GTEx Portal, eQTLGen Consortium (*p*-value<0.05)[22], RegulomeDB[23], YUE Lab (http://3dgenome.fsm.northwestern.edu/)[24], and the Ensemble Variant Effect Predictor[25–27].

Functional research on *TRPM2-AS* promoter

HELA, HEK-293 and HUVEC cells were cultured as per standard protocol (Human, *American Type Culture Collection* under Material Transfer Agreement, authenticated (microscopy/PCR), no mycoplasma contamination detected (anti-mycoplasma antibiotics, Normocin)). Total RNA was extracted using TRIzol™ Reagent (Cat. No. 15596026, Thermo scientific.), cDNA was synthesized using SuperScript™ III Reverse Transcriptase (Cat. No. 18080044, Invitrogen), and qPCR was done using iTaq™ Universal SYBR® Green Supermix (Cat. No. 1725121, Bio-Rad) as per manufacturer's protocol. Promoter sequence was PCR amplified using Phusion™ High-Fidelity DNA Polymerase (F530L, Thermo scientific.) from genomic DNA of one FinnDiane participant carrying rs753589764 minor allele, and one participant carrying none of the identified *TRPM2-AS* rare minor alleles; and cloned on pBV-Luc plasmid (Addgene plasmid #16539, gift from Bert Vogelstein). Transfection of promoter sequence carrying pBV-Luc reporter and renilla luciferase control plasmid was done using FuGENE® HD Transfection Reagent (Cat. No. E2311, Promega): Four technical repeats each transfection. Dual-Luciferase Reporter Assay (Cat. No. E1910, promega) was performed as per manufacturer's recommendations.

Single variant replication

We performed GWAS in individuals with T1D using score test (rvtests version 20190205)[8], and calculated statistical power with the genpwr R package 1.0.4[28]. Genotyping was successful for a varying number of individuals depending on the variant. We observed alternative allele carriers for six variants (**Table S20**) and performed single variant analyses similarly with score test. We analyzed one *LRRN1* variant with linear regression (stats R package 4.2.1) and without relatedness adjustment, because no alternative allele carriers were observed among individuals in the GWAS data i.e., kinship matrix.

We attempted general population replication from the FinnGen project release 6 (https://www.finngen.fi/en), and selected stroke phenotypes that best matched our definitions (**Table S23**).

Gene aggregate replication

We performed SKAT-O analyses in FinnDiane GWAS (r^2>0.80) for T1D specific replication with the GMMAT R package 1.3.2[29], by including also genotyped variants, and imputed missing data to mean (i.e., individuals with missing genotype dosage at a variant). We attempted general population replication from UK Biobank WES studies[30,31].

Sliding-window and regulatory region replication

We attempted T1D specific replication with GWAS data (r^2>0.80) using STAAR R package[14].

Supplementary Figures

Fig. S1: Clinical characteristics of individuals in WGS

A. Age, **B.** Diabetes duration, **C.** Age at diabetes onset, **D.** Calendar year of diabetes onset, **E.** Weighted mean HbA1c, **F.** DKD status.

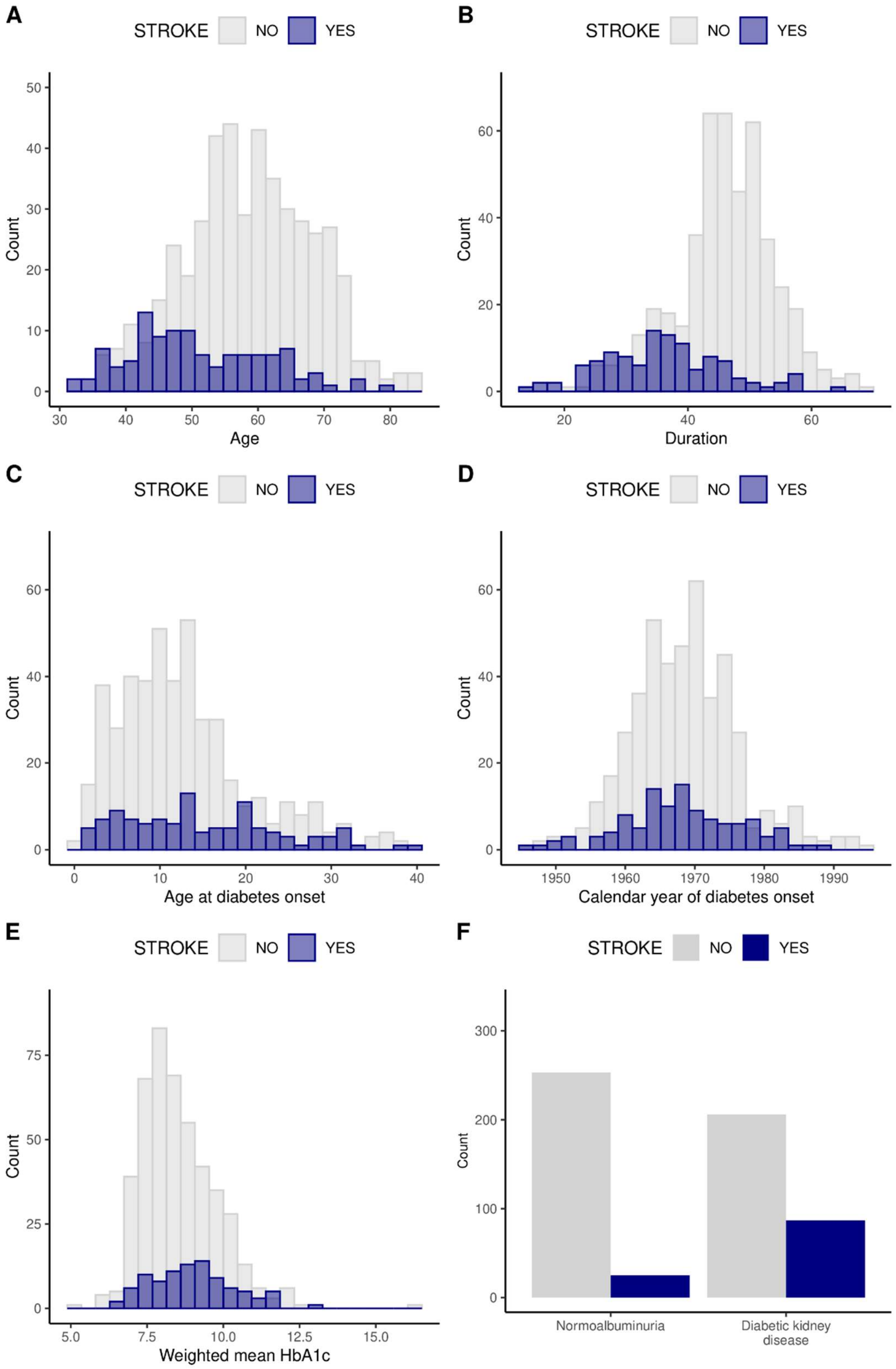

Fig. S2: Clinical characteristics of individuals in WES

A. Age, **B.** Diabetes duration, **C.** Age at diabetes onset, **D.** Calendar year of diabetes onset, **E.** Weighted mean HbA1c, **F.** DKD status.

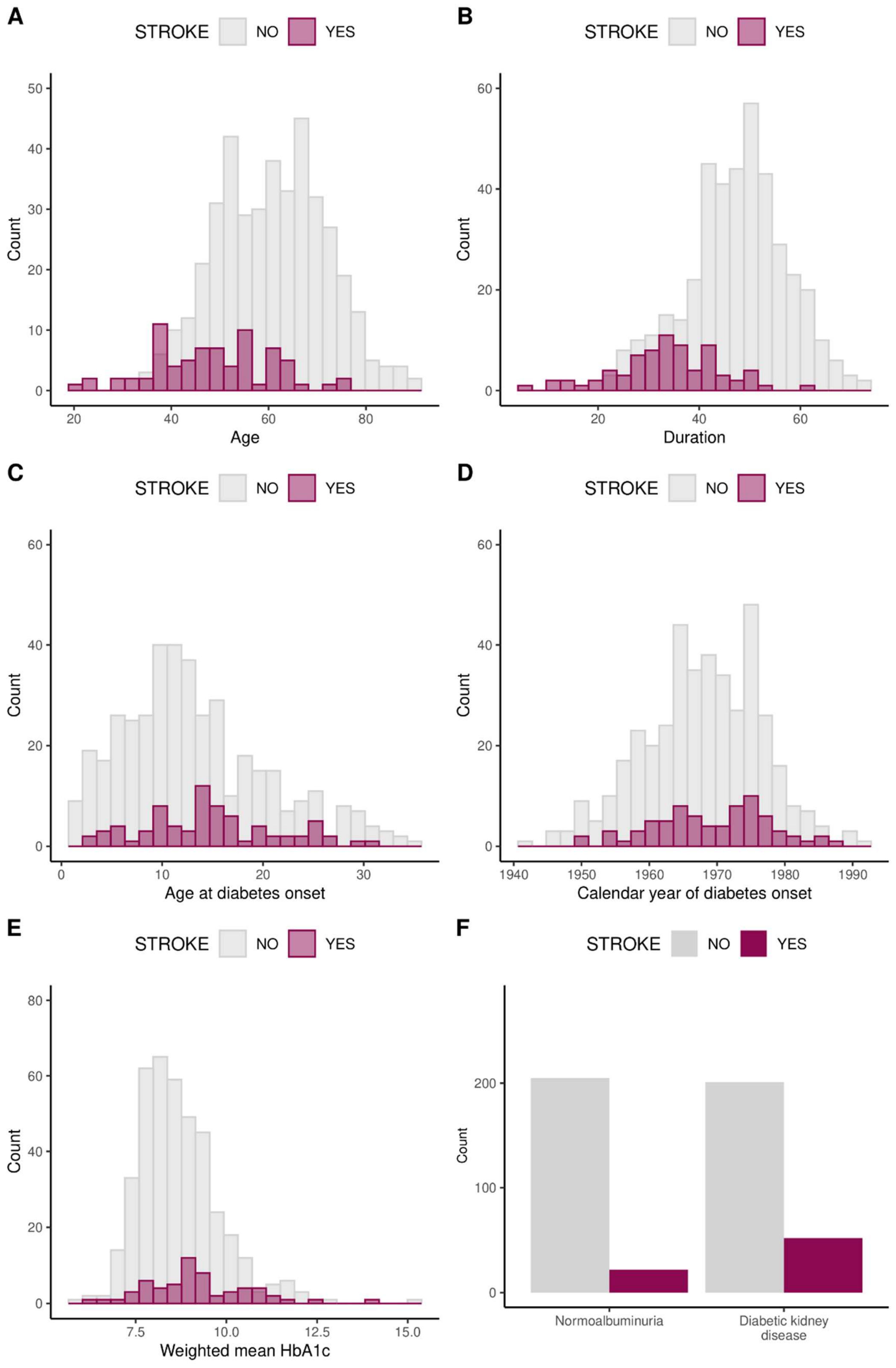

Fig. S3: Single variant analysis with DKD adjustment

Stroke single variant analysis Manhattan plot (Firth regression or score test fixe-effects meta-analysis).

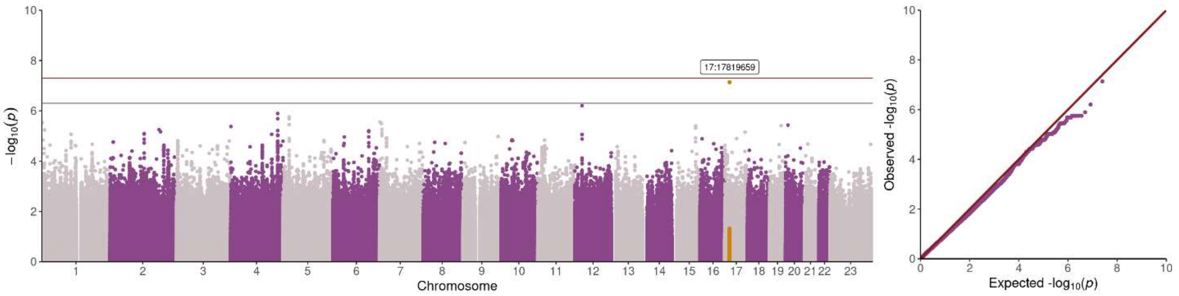

Fig. S4: SKAT-O minimal model Manhattan plot

A. Protein altering variant (PAV) ≤1%, **B.** PAV≤5%, **C.** Protein truncating variant (PTV) ≤1%, **D.** PTV≤5%.

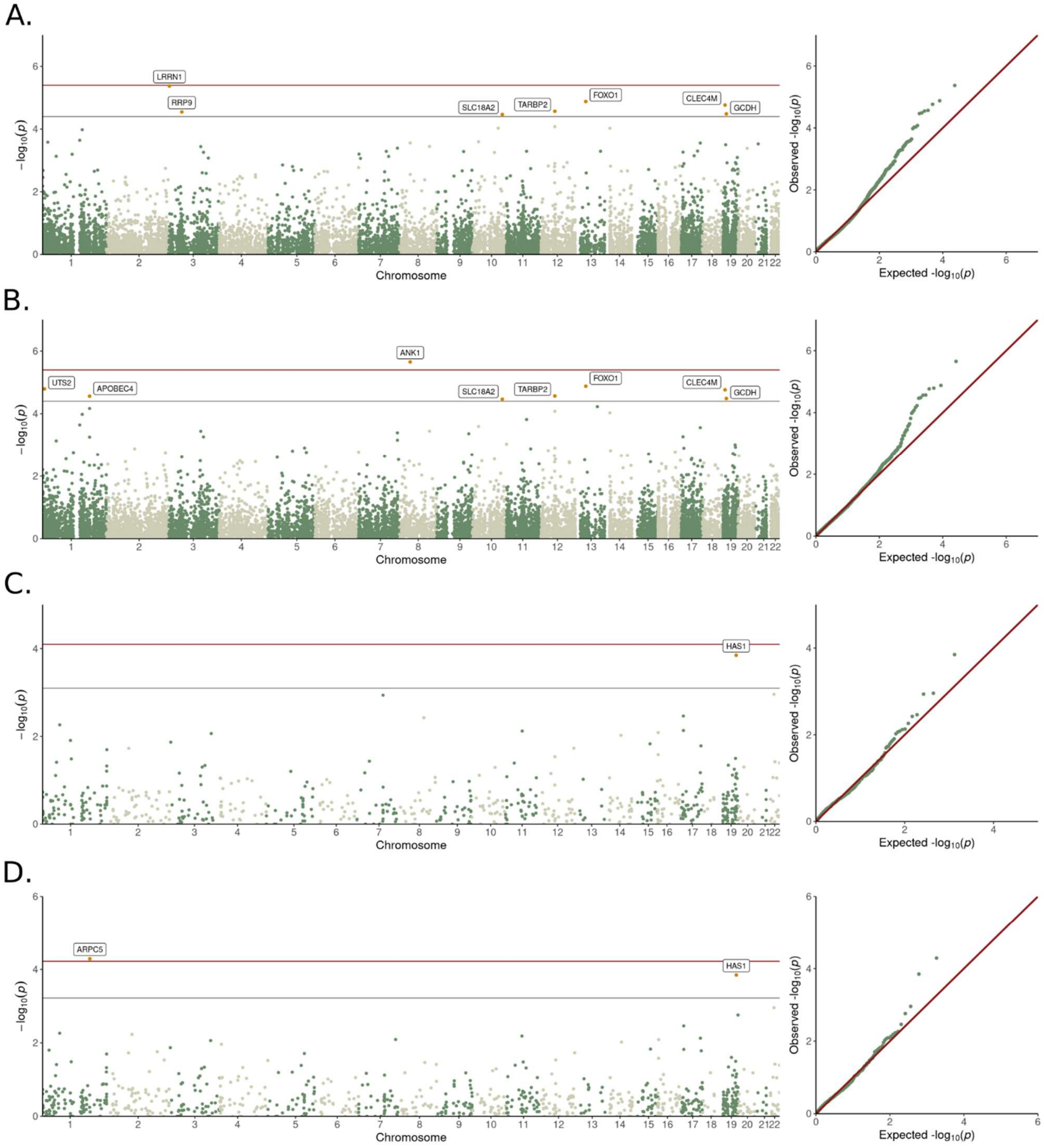

Fig. S5: SKAT-O with additional DKD adjustment Manhattan plot

A. Protein altering variant (PAV) ≤1%, **B.** PAV≤5%, **C.** Protein truncating variant (PTV) ≤1%, **D.** PTV≤5%.

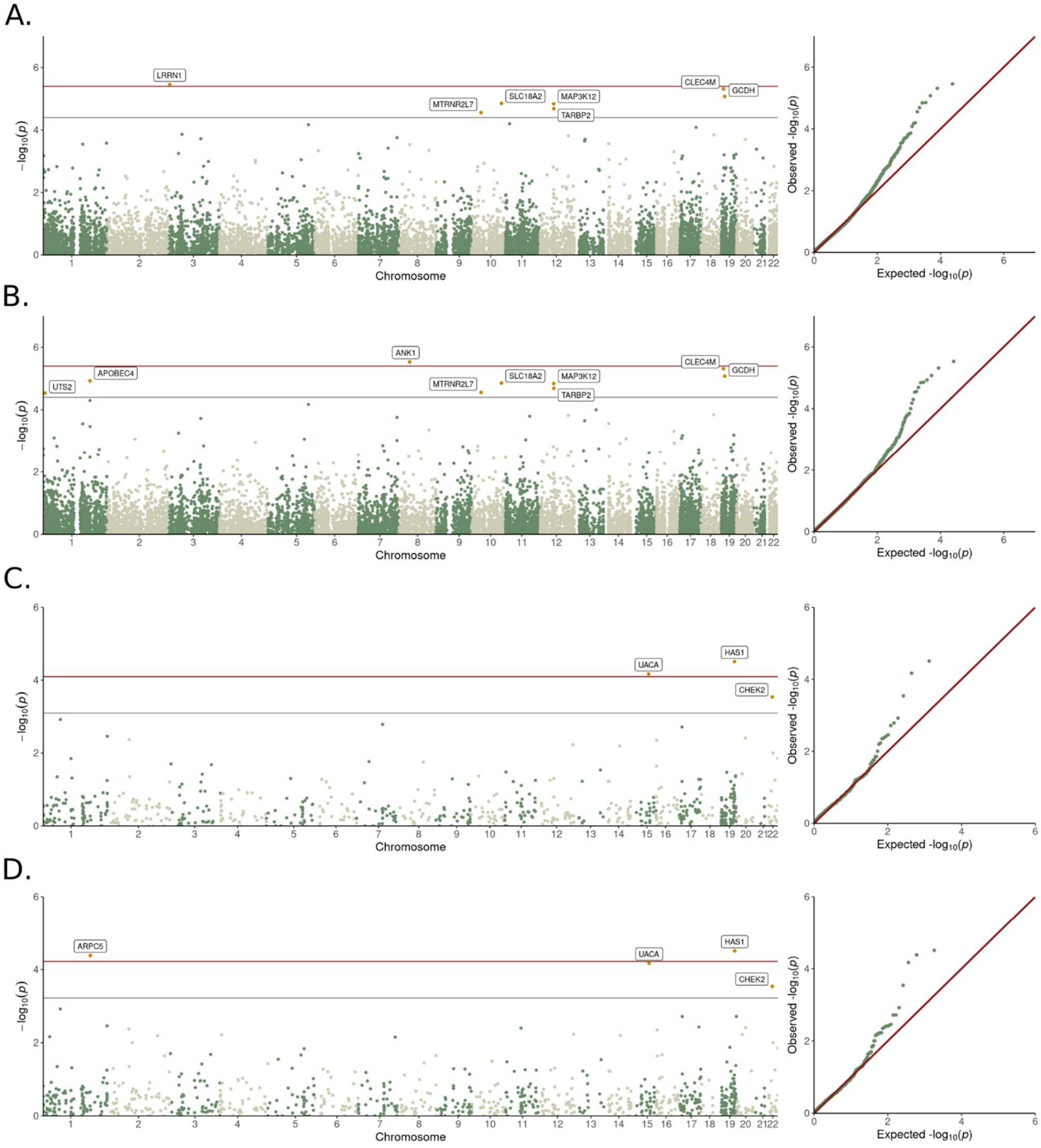

Fig. S6: *MAP3K12* and *TARBP2* regional plot

Chromosomal positions, genes and gene transcripts. Protein altering variants within SKAT-O tests are highlighted, and statistical significance are presented with the additional DKD adjustment.

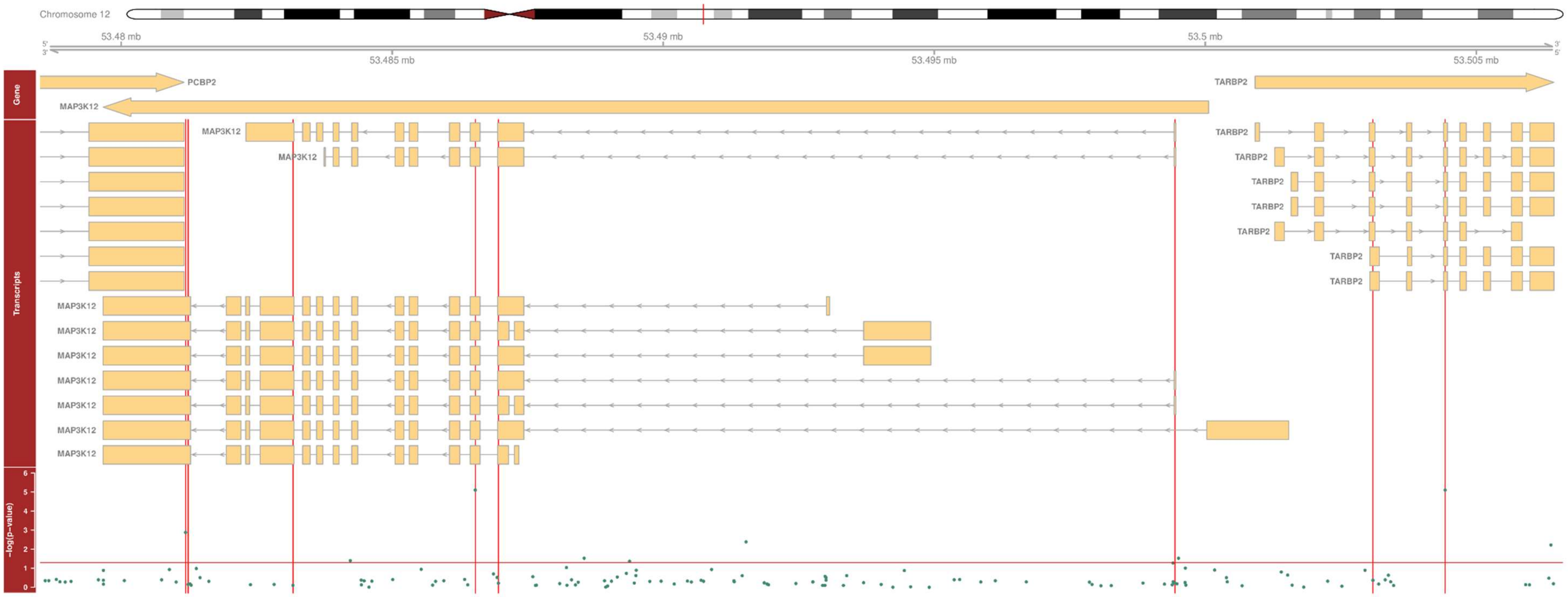

Fig. S7: Known Mendelian stroke-risk genes in T1D

A. SKAT-O *p*-value, **B.** $N_{variant}$ (cumulative minor allele count [CMAC]) in SKAT-O. Results are presented with minimal adjustment. We report associations for all Mendelian stroke-risk genes for which rare or low-frequency variant carriers were observed ($N_{variant}$>1); altogether 17 autosomal genes were investigated (*ABCC6*, *KRIT1*, *ADA2*, *COL3A1*, *COL4A1*, *COL4A2*, *COLGALT1*, *HTRA1*, *NOTCH3*, *RNF213*, *TREX1*, *CCM2*, *PDCD10*, *CTSA*, *APP*, *CST3*, *ITM2B*), of which 13 were succesfully analysed within our data.

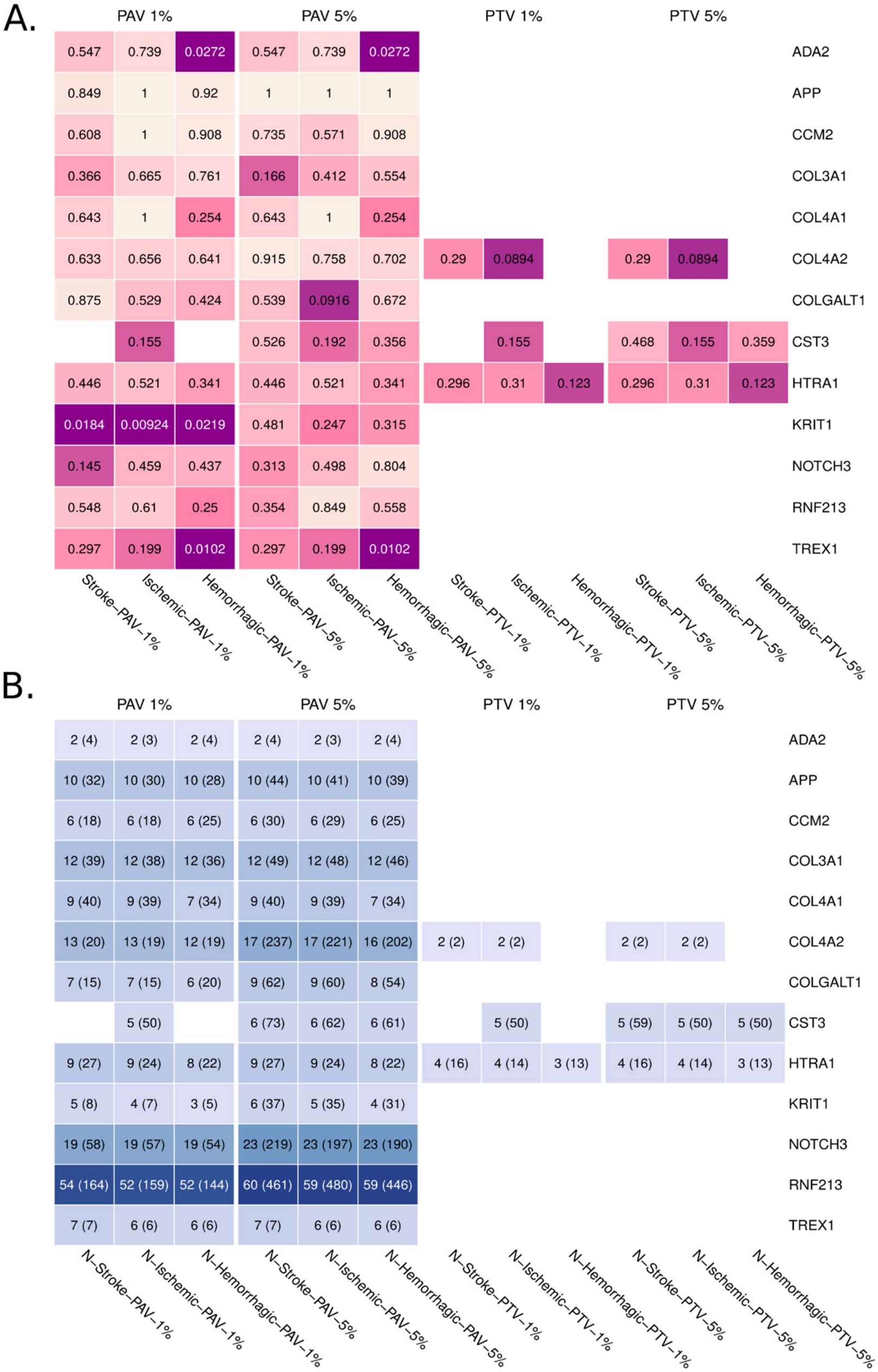

Fig. S8: Topologically associating domain (TAD) on 4q33-34.1[24]

A. Frontal lobe, **B.** Hippocampus. The region has been predicted to locate on the same TAD with *GALNTL6* promoter as well as *AADAT* and *MFAP3L* distal promoters. The identified windows are within 170,752,001-171,082,000 (highlighted with light blue); and the top variant is 4:170787127.

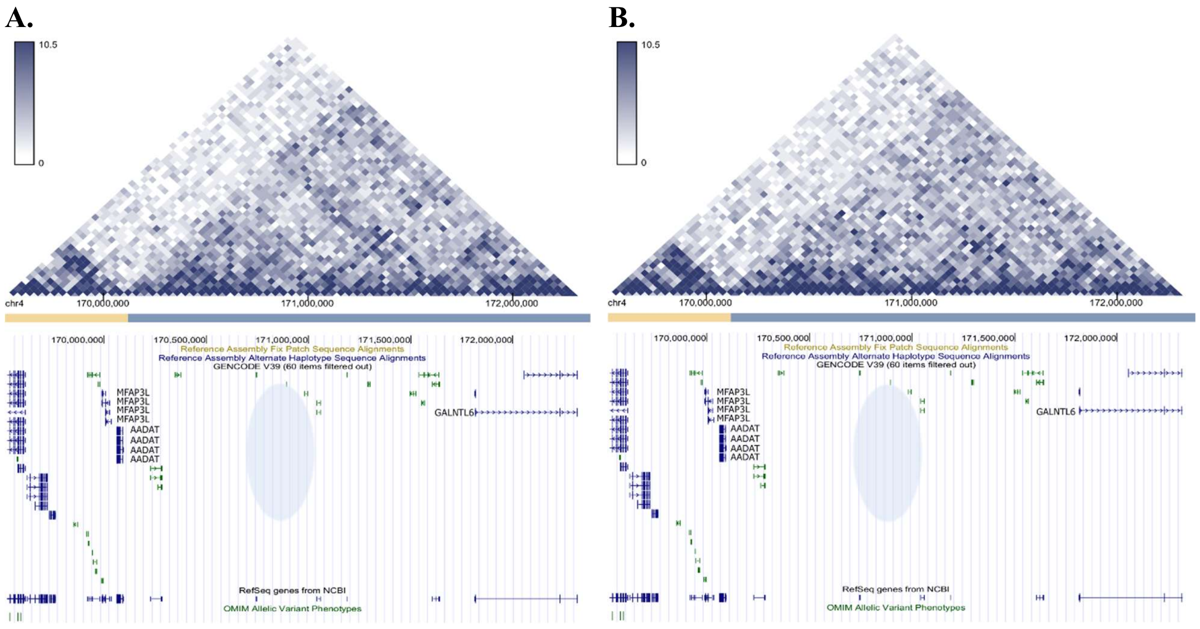

Fig. S9: Enhancer variant PCHi-C links[24]

A. *BDNF* in hippocampus, and **B.** An intronic sliding-window region, on *LINC01500*, links to *DACT1*.

A.

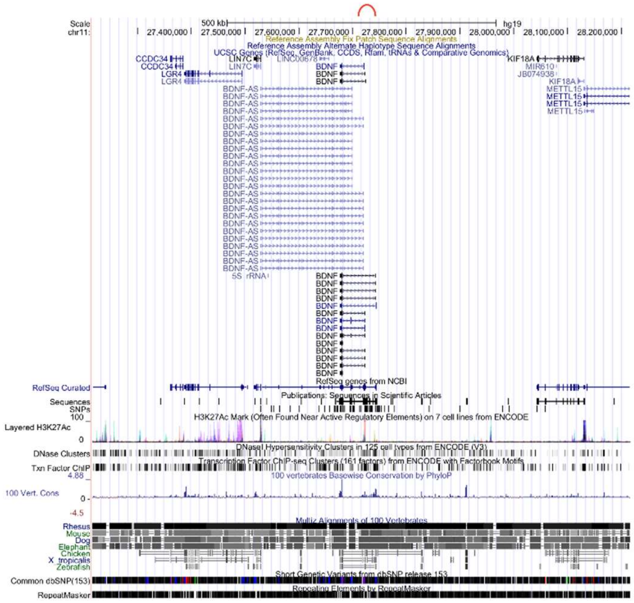

B.

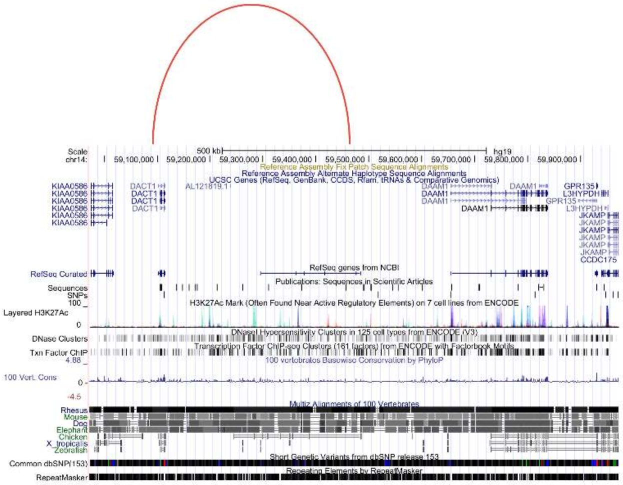

Fig. S10: Stroke enhancer association Manhattan plot

Variants weighted with PHRED scale minor allele frequencies. **A.** MAF≤5%, and **B.** MAF≤1%.

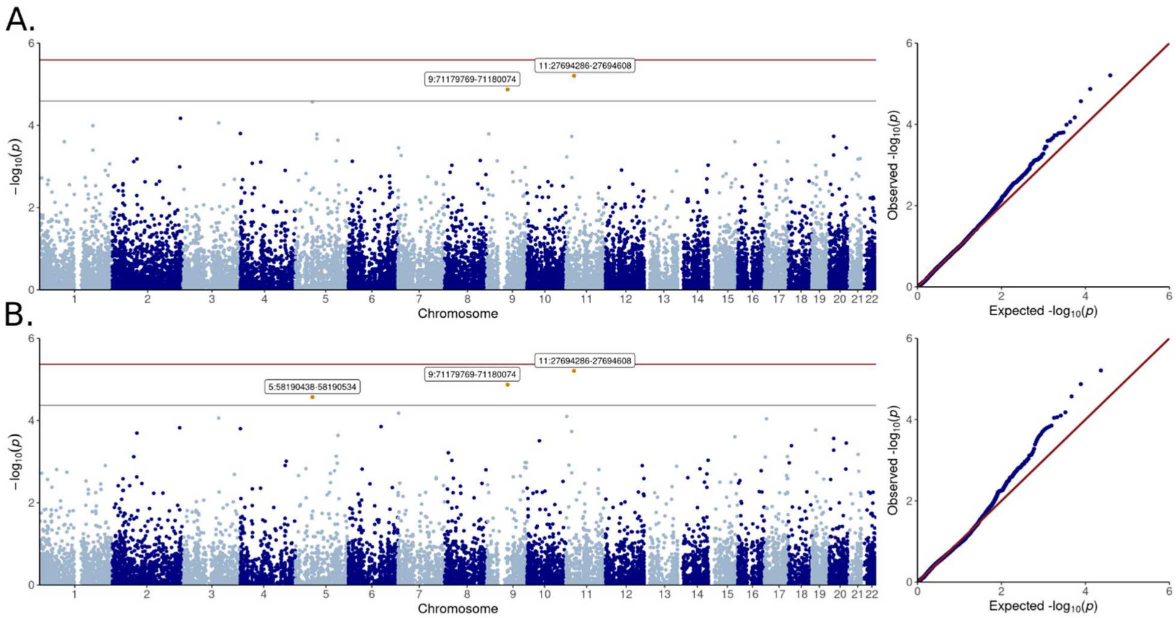

Fig. S11: Stroke promoter association Manhattan plot

Variants are weighted with PHRED scale minor allele frequencies. **A.** MAF≤5%, and **B.** MAF≤1%.

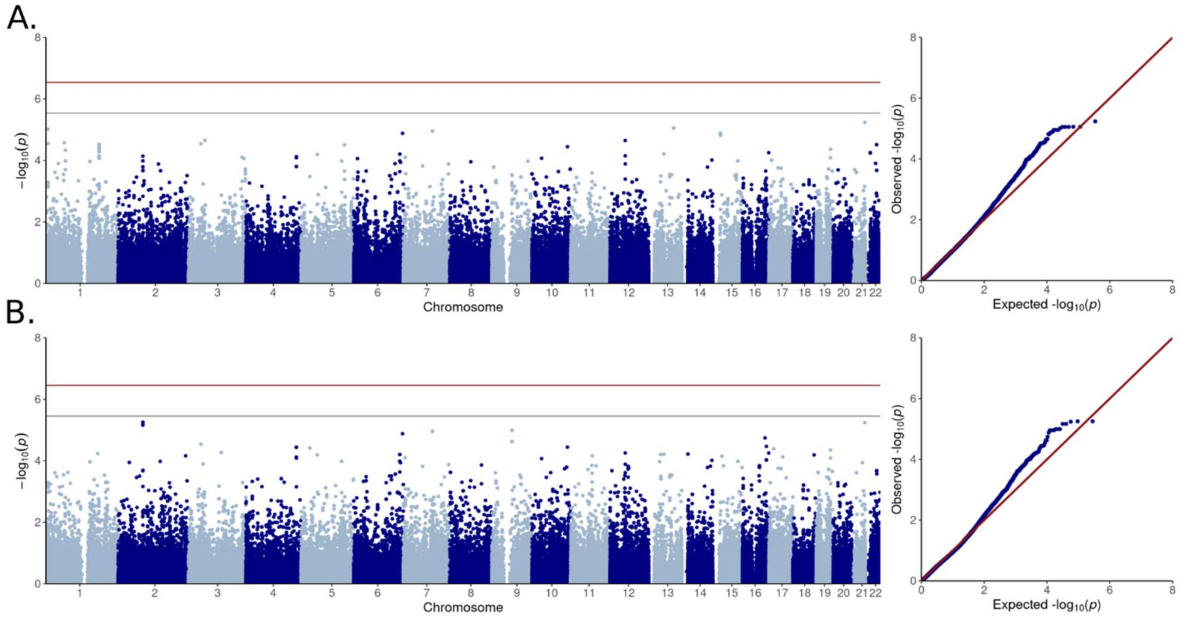

Fig. S12: Sequencing data processing pipeline (WES and WGS)

1. Genomes were sequenced with Illumina HiSeqX and Illumina HiSeq2000 next-generation sequencing, respectively. **2.** Genomes were trimmed with Trimmomatic 0.36 software[2], and read quality was assessed with FastQC software[32]. Next, we aligned reads with GATK's (4.0.1.1) Burrows-Wheeler Aligner (BWA), sorted and marked duplicate reads with Picard's SortSam and MarkDuplicates tools. We recalibrated bases by chromosome with GATK's VQSR and ApplyVQSR tools. **3.** Variants were called by sample with GATK's HaplotypeCaller using ERC mode, then WES and WGS were jointcalled into combined variant call format files with GATK's CombineGVCFs and GenotypeGVCF. 490 exomes passed quality control within WES: Two samples were excluded due to excess heterozygosity (>3 standard deviations from sample mean), one due to relatedness, one due to discordance with FinnDiane GWAS, and one due to being also whole genome sequenced. 583 samples passed QC within WGS: Five samples were excluded due to excess heterozygosity, eight due to failed percentage of mapped deduplicated reads (<91%), and three due to discordance with FinnDiane GWAS. We further performed quality control on individual variants.

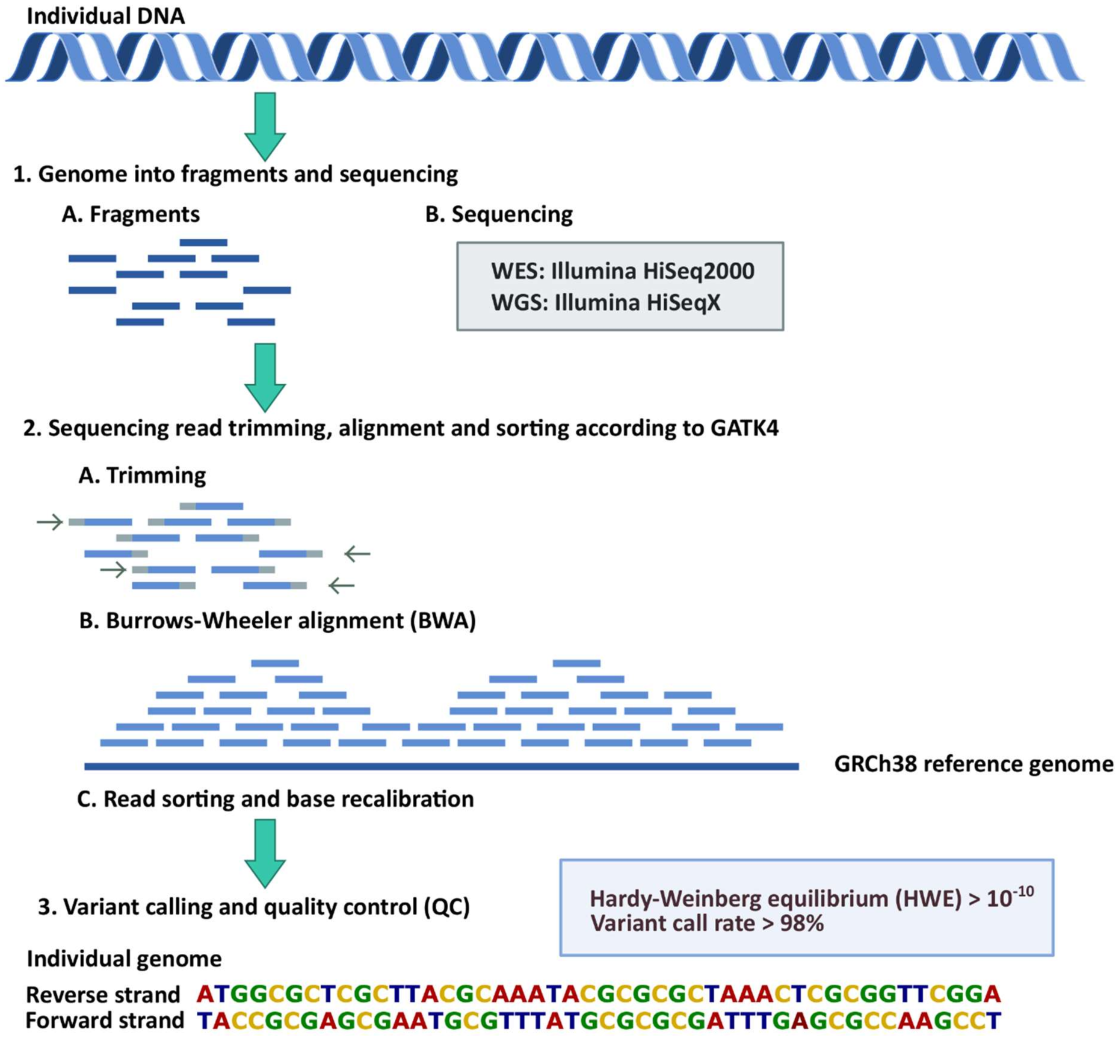

Fig. S13: GWAS replication data processing pipeline (FinnDiane)

The data had been previously processed to GRCh37 reference genome, importantly, variants had been called with zCall software[7] and low genotyping quality variants excluded. We have now shifted the genotyping positions from GRCh37 to GRCh38 with Picard's LiftoverVCF tool and merged the genotyping batches. Variants with high missingness (>2%), low HWE *p*-value ($<10^{-6}$), or minor allele count <3 were removed. Three individuals were excluded due to ambiguous gender, and six due to high genotype missingness rate (>5%) or heterozygosity (4 SDs from sample mean). Next, the chip genotyping data was pre-phased with Eagle 2.3.5 software[33], imputed to SISu v3 reference panel with Beagle 4.1 software[34], and annotated with SNPEff version 5 software[4]. FIMM HumGen Sequencing Informatics genotype imputation workflow v3.0 V2 was followed.

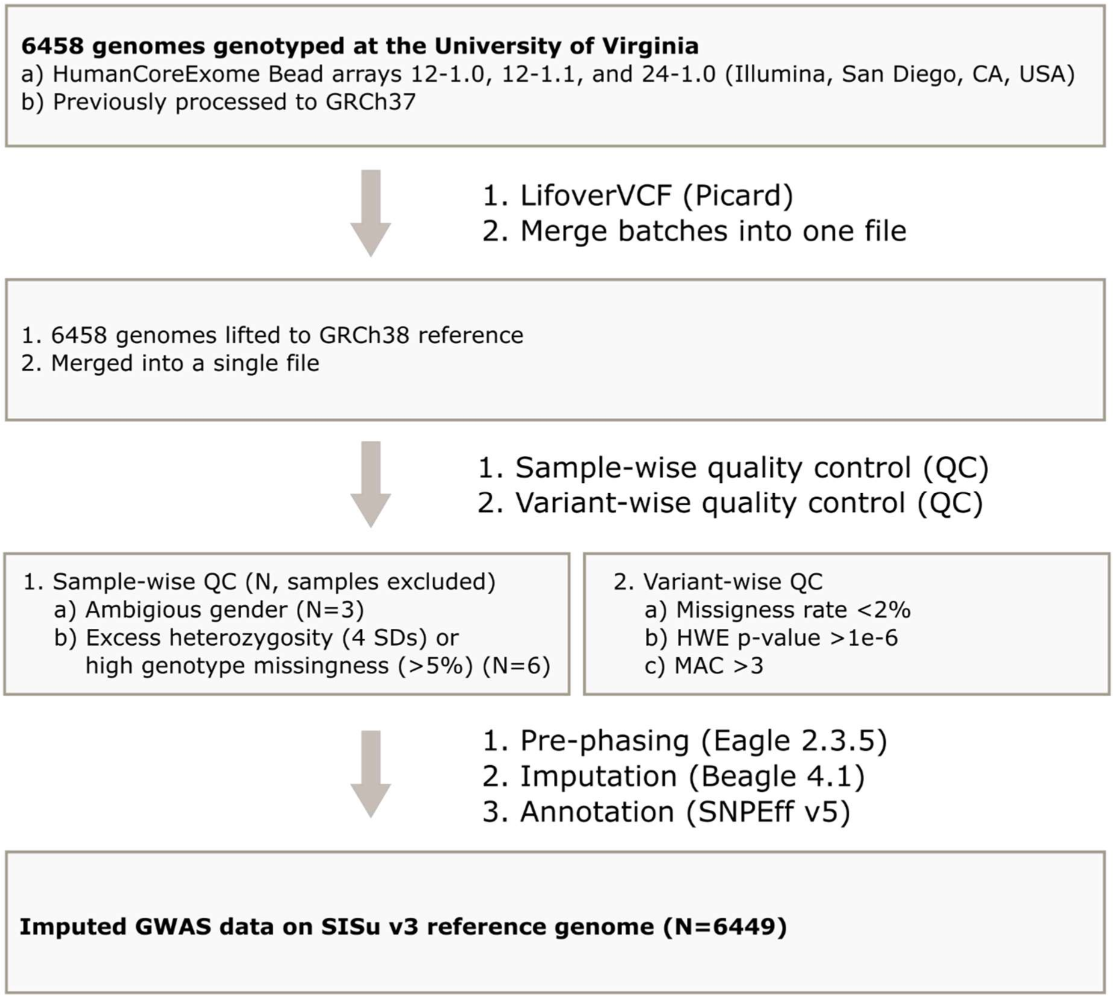

Fig. S14: Clinical characteristics of individuals in FinnDiane GWAS

GWAS replication within FinnDiane. **A.** Age, **B.** Diabetes duration, **C.** Age at diabetes onset, **D.** Calendar year of diabetes onset, **E.** Weighted mean HbA1c, **F.** DKD status.

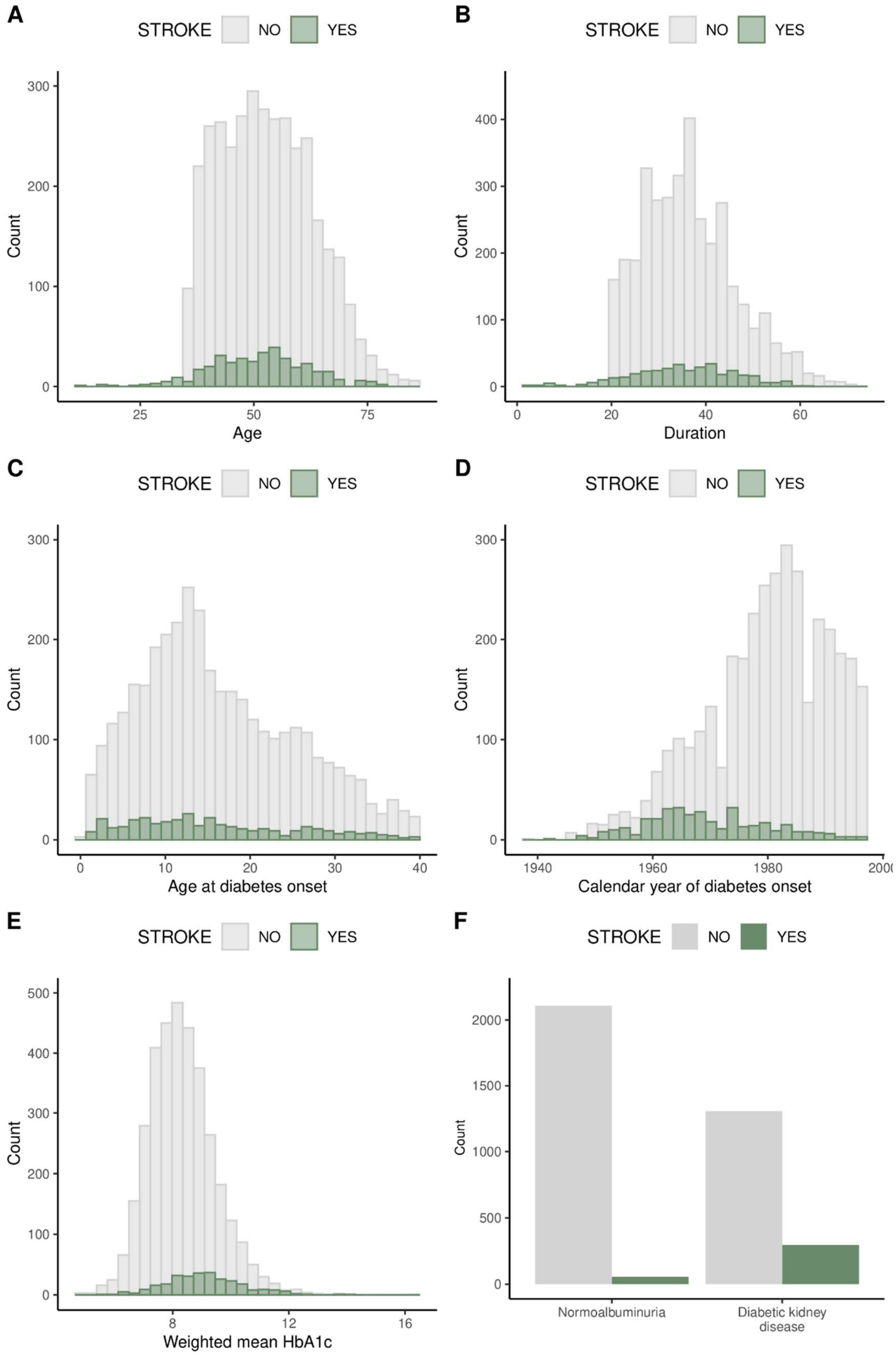

Fig. S15: Clinical characteristics of individuals in genotyping

Genotyping replication within FinnDiane. **A.** Age, **B.** Diabetes duration, **C.** Age at diabetes onset, **D.** Calendar year of diabetes onset, **E.** Weighted mean HbA1c, **F.** DKD status.

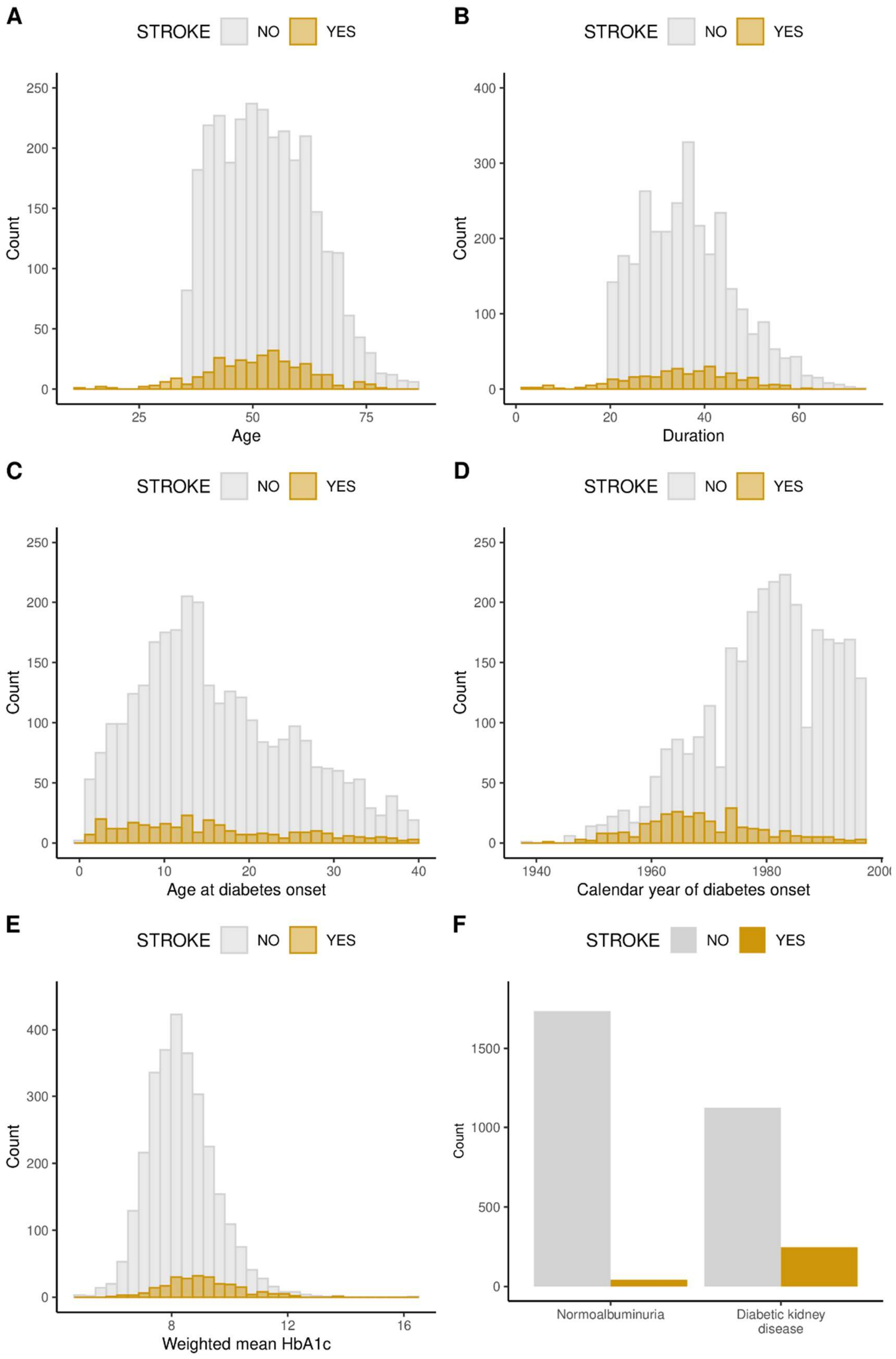

Fig. S16: Annotation PCA within functional classes (CADD)

A. Conservation, **B.** Epigenetics, **C.** microRNA, **D.** Mutation density, **E.** Protein function, **F.** Transcription factor, **G.** Proximity to transcription start- and end sites. Variance explained by the corresponding annotation principal component (aPC) is presented at Dimension 1.

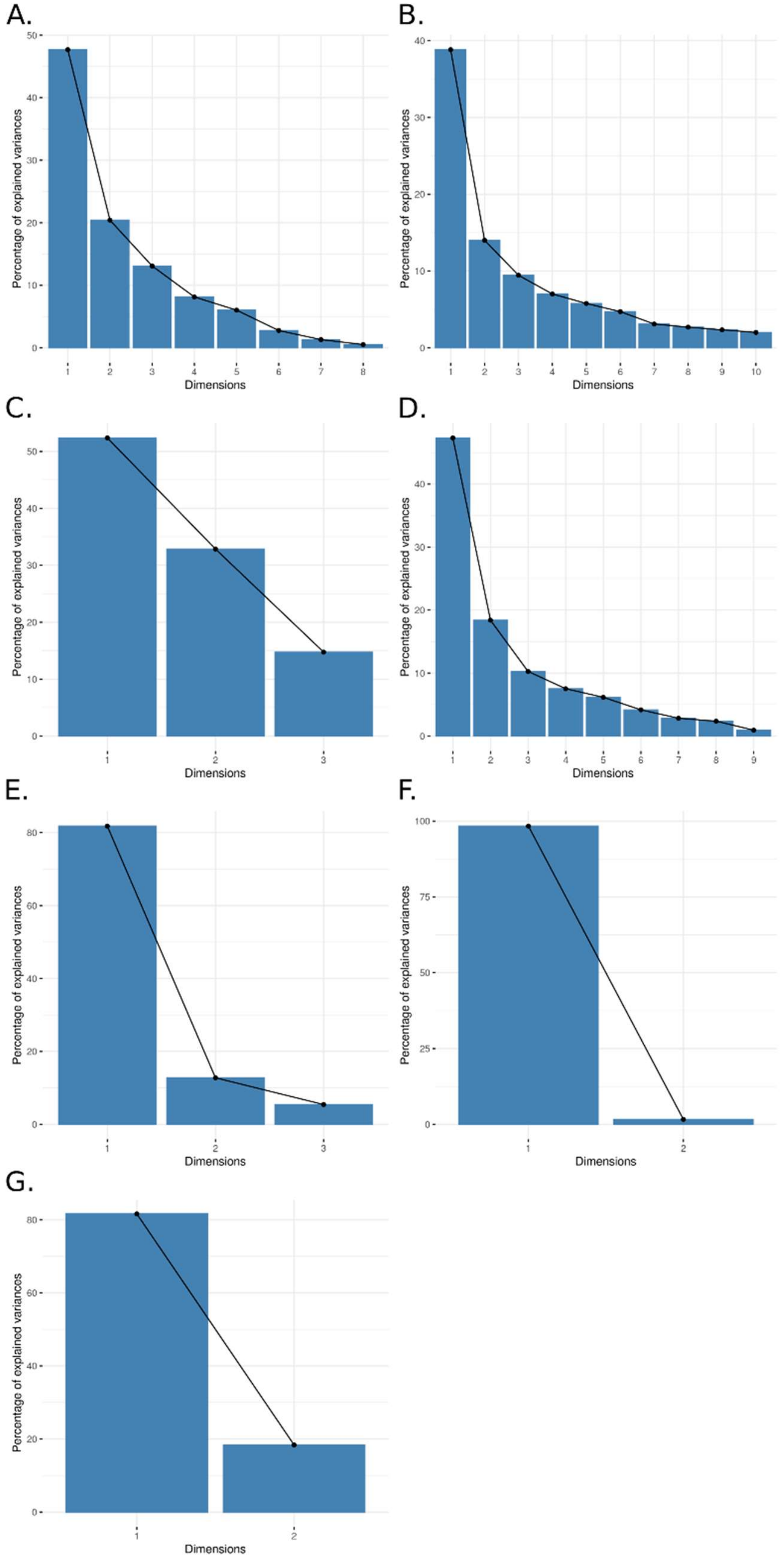

Fig. S17: Statistical power in replication

Statistical power to replicate low-frequency (MAF=5%, MAF=1%) and rare (MAF=0.5%, MAF=0.1%) variants with the number of patients in FinnDiane GWAS replication (N=3,945, N_{cases}=367) using different alpha levels: **A.** Nominal significance (0.05), **B.** Multiple testing corrected threshold (0.01), **C.** Multiple testing corrected threshold (0.005), **D.** Multiple testing corrected threshold (0.0005).

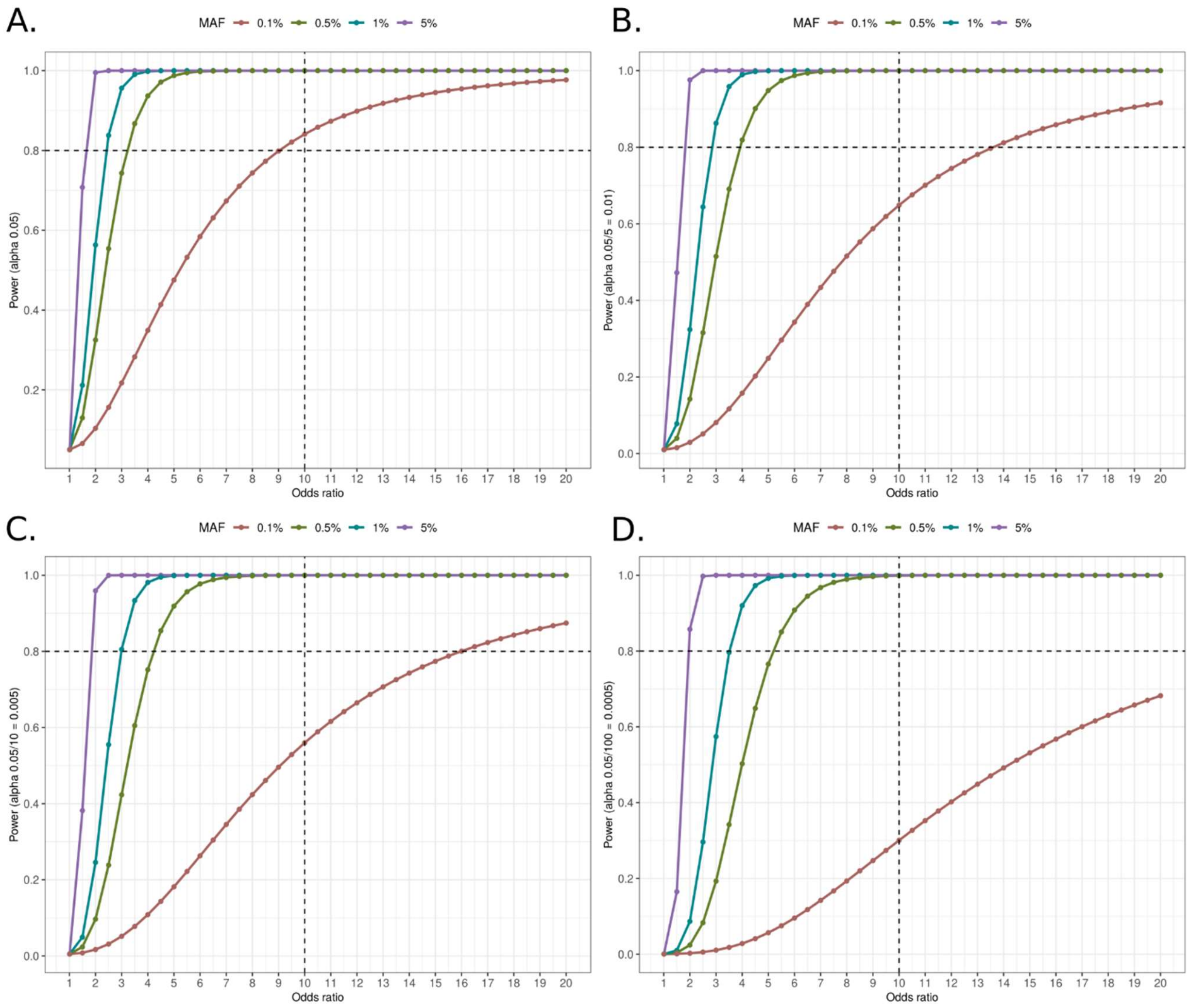

Fig. S18: PCA of sequencing data (WES and WGS)

A. Whole-exome sequencing data, **B.** Whole-genome sequencing data.

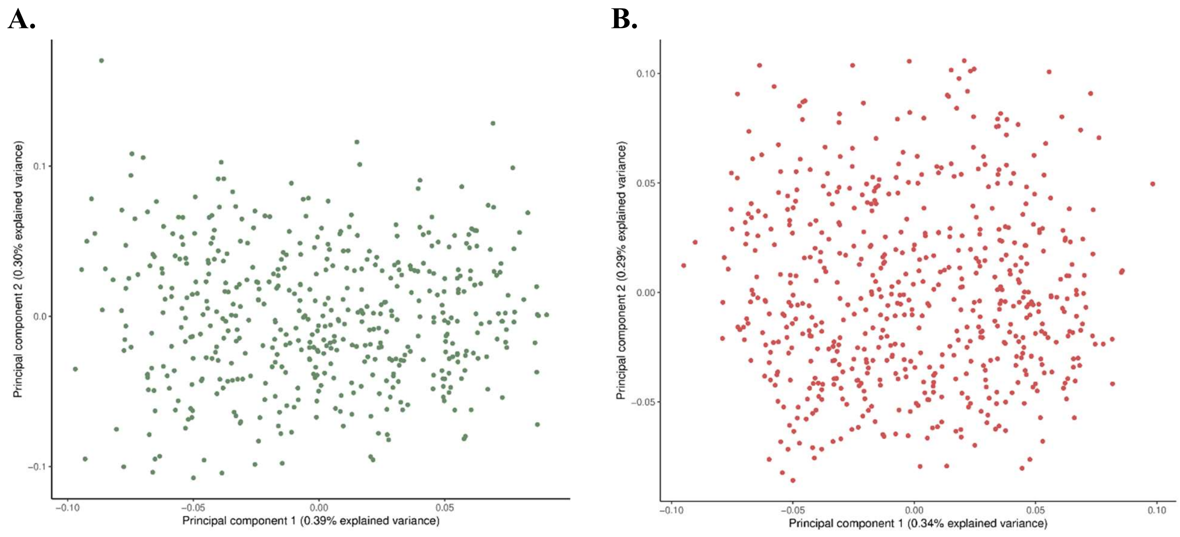

Fig. S19: *TRPM2-AS* expression in HELA, HEK-293 and HUVEC cell lines

Uncropped version of the gel image in **Figure 6B**.

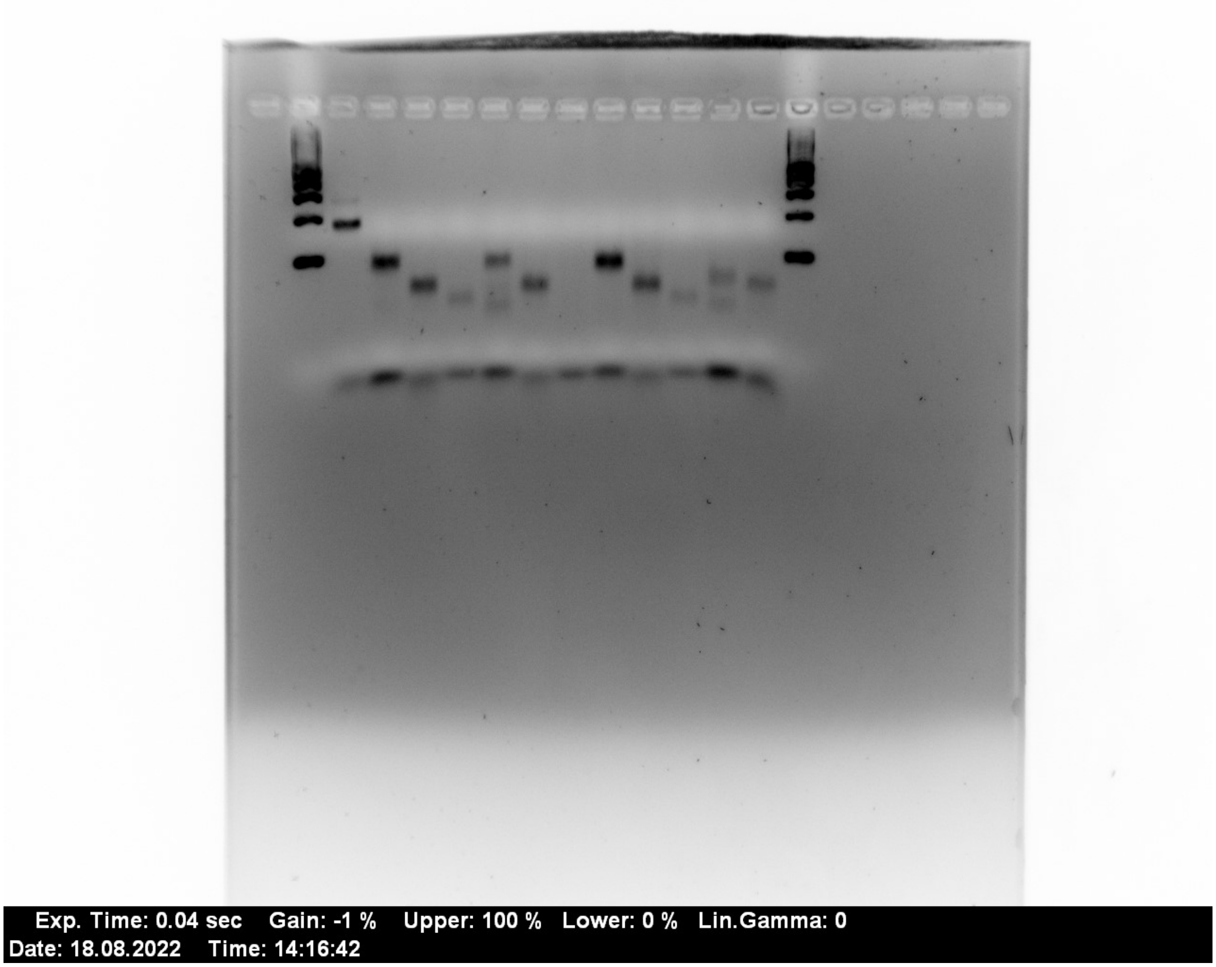

Supplementary Tables

Table S1: Stroke subtype sequencing data clinical characteristics

A. Ischemic stroke, **B**. Hemorrhagic stroke. Weighted mean HbA1c is calculated until the stroke event or the end of follow-up. DKD = End-stage renal disease, macro- or microalbuminuria. Mean (SD), *Median (IQR). Student's t-test, Wilcoxon signed rank test or Fisher's exact test.

A.

	WES			WGS		
	**Cases**	**Controls**	***p*-value**	**Cases**	**Controls**	***p*-value**
N	49	406		64	459	
CVD death <2017 (yes/no, yes-%)	22/27 (45%)	66/340 (16%)	1.20×10^{-5}	31/33 (48%)	83/376 (18%)	3.93×10^{-7}
Sex (male/female, %-males)	26/23 (53%)	182/224 (45%)	0.29	45/19 (70%)	236/223 (51%)	0.0048
Age	49.40 (12.46)	60.69 (11.13)	1.12×10^{-7}	51.49 (10.12)	58.70 (9.61)	7.63×10^{-7}
T1D Duration	33.63 (10.31)	47.49 (9.84)	1.45×10^{-12}	36.63 (9.15)	46.00 (8.14)	2.59×10^{-11}
Calendar year of diabetes onset*	1971 (11)	1968 (11)	0.21	1967.5 (12)	1969 (9)	0.41
T1D onset age	15.77 (7.26)	13.21 (7.27)	0.023	14.86 (8.79)	12.69 (7.75)	0.065
Weighted mean HbA1c*	9.1 (2.11)	8.51 (1.40)	0.015	8.86 (1.74)	8.34 (1.57)	0.0062
HbA1c count*	23 (31)	29 (26)	0.039	19 (28.75)	29 (30)	0.068
DKD (yes/no, yes-%)	35/14 (71%)	201/205 (50%)	0.0039	53/11 (83%)	206/253 (45%)	6.76×10^{-9}

B.

	WES			WGS		
	**Cases**	**Controls**	***p*-value**	**Cases**	**Controls**	***p*-value**
N	22	406		26	459	
CVD death <2017 (yes/no, yes-%)	12/10 (55%)	66/340 (16%)	8.46×10^{-5}	16/10 (62%)	83/376 (18%)	2.75×10^{-6}
Sex (male/female, %-males)	13/9 (59%)	182/224 (45%)	0.27	19/7 (73%)	236/223 (51%)	0.042
Age	45.98 (10.97)	60.69 (11.13)	2.82×10^{-6}	49.42 (11.34)	58.7 (9.61)	0.00035
T1D Duration	32.96 (11.30)	47.49 (9.84)	5.24×10^{-6}	36.72 (10.81)	46.00 (8.14)	0.00020
Calendar year of T1D onset*	1968.5 (11.25)	1968 (11)	0.90	1968 (12.5)	1969 (9)	0.85
T1D onset age	13.02 (5.02)	13.21 (7.27)	0.87	12.7 (8.37)	12.69 (7.75)	1.00
Weighted mean HbA1c*	8.99 (0.45)	8.51 (1.40)	0.050	8.92 (1.91)	8.34 (1.57)	0.15
HbA1c count*	13.5 (12.75)	29 (26)	0.010	22 (25.75)	29 (30)	0.092
DKD (yes/no, yes-%)	15/7 (68%)	201/205 (50%)	0.12	20/6 (77%)	206/253 (45%)	0.0019

Table S16: Stroke ICD codes in Finnish registry data

For patients in sequencing cohort without data verified by neurologists (N_{WGS}=27, N_{WES}=2), we considered only the registry data as follows: Stroke was defined as *Stroke, severe* (I60, I61, I62, I63, I64, 430, 431, 432, 433, 434), and we excluded *Stroke, mild* (I65, I66, I67, I68, I69, 435, 436, 437, 438, TIA G45) from controls in order to ensure a clean phenotype (WGS N=5, WES N=0). In T1D specific replication data (GWAS and genotyping), registry-based stroke events were partly verified by neurologists, and whenever the verified data were unavailable, we defined stroke as *Stroke, severe* (I60, I61, I62, I63, I64, 430, 431, 432, 433, 434). Similarly, *Stroke, mild* (I65, I66, I67, I68, I69, 435, 436, 437, 438, TIA G45) were excluded from controls. Controls were followed until death or the end of 2017.

**Stroke, severe**		**Stroke mild**	
**Explanation**	**ICD code**	**Explanation**	**ICD code**
Subarachnoid hemorrhage	I60	Occlusion and stenosis of precerebral arteries not resulting in cerebral infarction	I65
Intracerebral hemorrhage	I61	Occlusion and stenosis of cerebral arteries not resulting in cerebral infarction	I66
Other nontraumatic intracranial haemorrhage	I62	Other cerebrovascular diseases	I67
Cerebral infarction	I63	Cerebrovascular disorders in diseases classified elsewhere	I68
Stroke, not specified as hemorrhage or infarction	I64	Sequellae of cerebrovascular disease	I69
Subarachnoid hemorrhage	430	Transient cerebral ischemia	435
Intracerebral hemorrhage	431	Acute but ill-defined cerebrovascular disease	436
Other and unspecified intracranial hemorrhage	432	Other and ill-defined cerebrovascular disease	437
Occlusion and stenosis of precerebral arteries	433	Late effects of cerebrovascular disease	438
Occlusion of cerebral arteries	434	Transient cerebral ischemic attacks and related syndromes	TIA G45

Table S17: GWAS replication clinical characteristics (FinnDiane)

Out of the stroke cases, 206 were verified by trained neurologists and 161 were based on registry data. Weighted mean HbA1c is calculated until the stroke event or the end of follow-up. DKD = End-stage renal disease, macro- or microalbuminuria. Mean (SD), *Median (IQR). Student's t-test, Wilcoxon signed rank test or Fisher's exact test.

	Stroke		
	**Cases**	**Controls**	***p*-value**
N	367	3578	
Hemorrhagic/Ischemic	40/164		
CVD death <2017 (yes/no, yes-%)	160/207 (44%)	232/3346 (6.5%)	2.70×10^{-74}
Sex (male/female, %-males)	225/142 (61%)	1819/1759 (51%)	0.00015
Age	51.02 (11.00)	52.83 (10.28)	0.0028
T1D Duration	35.14 (11.20)	36.55 (9.93)	0.021
Calendar year of T1D onset*	1968 (15)	1981 (15)	8.53×10^{-60}
T1D onset age*	14.21 (14.88)	14.48 (13.22)	0.23
Weighted mean HbA1c	9.10 (1.33)	8.33 (1.14)	2.94×10^{-19}
HbA1c count*	17 (27)	22 (27)	0.0024
DKD (yes/no, yes-%)	296/56 (84%)	1305/2108 (38%)	7.24×10^{-64}

Table S18: GWAS stroke subtype replication clinical characteristics

Weighted mean HbA1c is calculated until the stroke event or the end of follow-up. DKD = End-stage renal disease, macro- or microalbuminuria. Mean (SD), *Median (IQR). Student's t-test, Wilcoxon signed rank test or Fisher's exact test.

	Hemorrhagic stroke			Ischemic stroke		
	**Cases**	**Controls**	**p-value**	**Cases**	**Controls**	**p-value**
	40	3578		164	3578	
CVD death <2017 (yes/no, yes-%)	20/20 (50%)	232/3346 (6.5%)	1.36×10^{-13}	72/92 (44%)	232/3346 (6.5%)	7.18×10^{-38}
Sex (male/female, %-males)	27/13 (68%)	1819/1759 (51%)	0.039	108/56 (66%)	1819/1759 (51%)	0.00016
Age	49.96 (7.31)	52.83 (10.28)	0.019	52.23 (10.85)	52.83 (10.28)	0.49
T1D Duration	35.84 (9.37)	36.55 (9.93)	0.64	35.09 (10.53)	36.55 (9.93)	0.083
Calendar year of T1D onset*	1965.5 (13.25)	1981 (15)	1.00×10^{-10}	1968 (13.25)	1981 (15)	4.47×10^{-32}
T1D onset age*	11.33 (11.52)	14.48 (13.22)	0.072	15.42 (15.63)	14.48 (13.22)	0.404
Weighted mean HbA1c	8.8 (1.43)	8.33 (1.14)	0.062	9.04 (1.18)	8.33 (1.14)	5.73×10^{-10}
HbA1c count*	8 (13.5)	22 (27)	0.00046	16 (27.25)	22 (27)	0.0040
DKD (yes/no, yes-%)	35/4 (90%)	1305/2108 (38%)	4.04×10^{-11}	131/25 (84%)	1305/2108 (38%)	1.88×10^{-30}

Table S19: FinnDiane genotyping clinical characteristics

Out of the stroke cases, 201 were verified by trained neurologists and 102 were based on registry data. Weighted mean HbA1c is calculated until the stroke event or the end of follow-up. DKD = End-stage renal disease, macro- or microalbuminuria. Mean (SD), *Median (IQR). Student's t-test, Wilcoxon signed rank test or Fisher's exact test.

	**Stroke**		
	**Cases**	**Controls**	***p*-value**
N	303	2960	
Hemorrhagic/Ischemic	39/160		
CVD death <2017 (yes/no, yes-%)	131/172 (43%)	191/2769 (6.5%)	6.45×10^{-61}
Sex (male/female, %-males)	185/118 (61%)	1508/1452 (51%)	0.00088
Age	50.91 (11.07)	52.92 (10.42)	0.0028
T1D Duration	35.19 (11.26)	36.60 (10.10)	0.037
Calendar year of T1D onset*	1968 (14)	1981 (15)	2.35×10^{-52}
T1D onset age*	13.76 (15.50)	14.39 (13.20)	0.14
Weighted mean HbA1c	9.03 (1.25)	8.35 (1.16)	8.06×10^{-15}
HbA1c count*	16 (27)	23 (25)	0.00030
DKD (yes/no, yes-%)	247/43 (85%)	1123/1735 (39%)	3.77×10^{-53}

Table S20: Genotyped variants in FinnDiane

Variants were genotyped for 3,600 individuals (including the positive control), although replication by genotyping entailed only individuals within FinnDiane GWAS data – thus, the kinship matrix – and those with available stroke phenotype and fulfilled control criteria (i.e., age >35 years, diabetes duration >20 years, and no mild strokes in registry data, if data verified neurologists not available) (N=3,263). One rare variant carrier was selected from sequencing data to be the positive control, and the patient was later excluded from the replication analyses.

Variant	REF	ALT	rsnumber	Sequenced	ALT AC[†]
1:183648589*	GT	G	.	OK	0
1:183653304*	T	C	rs1361824345	OK	0
15:70679607	C	A	rs185763236	OK	≤3
17:17819659	G	A	rs114001633	OK	16
19:12897009*	G	A	rs761135089	OK	0
19:51716967	C	T	rs1396240967	OK	0
19:7766121*	A	G	rs144783051	OK	10
19:7767549	C	T	rs752738017	OK	≤3
3:3845194	C	T	rs142381203	OK	≤3[‡]
3:3845554	C	T	rs141825989	OK	≤3
3:3845966	C	T	rs747455683	OK	0
19:7766659	G	A	rs140767813	OK	≤3

*Added for runs 4-10.

†Alternative allele count in all genotyped individuals (excluding the positive control, N_{total}=3,599).

‡Analysed in R with linear regression due to no alternative allele carriers after kinship matrix adjustment criteria (N=3,263)

Table S21: Variant type classification for SKAT-O

	Protein altering variants (PAV)	Protein truncating variants (PTV)
**Variant type**	5 prime UTR premature start codon gain variant	start lost
	5 prime UTR truncation & exon loss variant	stop gained
	bidirectional gene fusion	stop lost
	gene fusion	bidirectional gene fusion
	conservative inframe deletion	gene fusion
	conservative inframe insertion	frameshift variant
	disruptive inframe deletion	exon loss variant
	disruptive inframe insertion	splice acceptor variant
	start lost	splice donor variant
	stop gained	
	stop lost	
	exon loss variant	
	frameshift variant	
	missense variant	
	splice acceptor variant	
	splice donor variant	
	structural interaction variant	

Table S22: CADD functional annotations within annotation classes

Listed are CADD annotation scores, which we used in calculation of annotation principal components (aPC)[15] for STAAR-O sliding-window analyses.

**Functional score**	**CADD annotations included**
**a-PC-Epigenetics**	GC, CpG, EncodeH3K4me1-max, EncodeH3K4me2-max, EncodeH3K4me3-max, EncodeH3K9ac-max, EncodeH3K9me3-max, EncodeH3K27ac-max, EncodeH3K27me3-max, EncodeH3K36me3-max, EncodeH3K79me2-max, EncodeH4K20me1-max, EncodeH2AFZ-max, RemapOverlapTF, RemapOverlapCL, minDistTSS, minDistTSE
**aPC-TF**	RemapOverlapTF, RemapOverlapCL
**aPC-Conservation**	GerpN, GerpS, priPhCons, mamPhCons, verPhCons, priPhyloP, mamPhyloP, verPhyloP
**aPC-Protein-function**	SIFTval, PolyPhenVal, Grantham
**aPC-microRNA**	targetScan, mirSVR.Score, mirSVR.E
**aPC-Mutation-density**	Freq100bp, Rare100bp, Sngl100bp, Freq1000bp, Rare1000bp, Sngl1000bp, Freq10000bp, Rare10000bp, Sngl10000bp
**aPC-TES-TSS-proximity**	minDistTSS, minDistTSE

Table S23: FinnGen replication ICD codes (GWAS)

Stroke, including SAH, is the main general population replication phenotype. We performed replication also with additional FinnGen stroke phenotypes, most importantly; ischemic stroke (*Ischemic stroke, excluding all hemorrhages*) and hemorrhages (*Nontraumatic intracranial hemorrhages*), but also (*Stroke, excluding SAH*).

**Phenotype**	**Name**	**ICD codes**
Nontraumatic intracranial hemorrhage	I9_INTRACRA	I60-61, 430-431
Ischemic stroke, excluding all hemorrhages	I9_STR_EXH	I63-64, 433-434, 436
Stroke, excluding SAH	I9_STR	I61, I63-64, 431, 433-434, 436
Stroke, including SAH	I9_STR_SAH	I60-61, I63-64, 430-431, 433-434, 436

Table S24: Physicians and nurses at health care centers participating in the collection of FinnDiane patients

FinnDiane Study Centers	Physicians and nurses
**Anjalankoski Health Centre**	S. Koivula, T. Uggeldahl
**Central Finland Central Hospital, Jyväskylä**	T. Forslund, A. Halonen, A. Koistinen, P. Koskiaho, M. Laukkanen, J. Saltevo, M. Tiihonen
**Central Hospital of Åland Islands, Mariehamn**	M. Forsen, H. Granlund, A-C. Jonsson, B. Nyroos
**Central Hospital of Kanta-Häme, Hämeenlinna**	P. Kinnunen, A. Orvola, T. Salonen, A. Vähänen
**Central Hospital of Länsi-Pohja, Kemi**	H. Laukkanen, P. Nyländen, A. Sademies
**Central Ostrabothnian Hospital District, Kokkola**	S. Anderson, B. Asplund, U. Byskata, P. Liedes, M. Kuusela, T. Virkkala
**City of Espoo Health Centre**	
**Espoonlahti**	A. Nikkola, E. Ritola
**Tapiola**	M. Niska, H. Saarinen
**Samaria**	E. Oukko-Ruponen, T. Virtanen
**Viherlaakso**	A. Lyytinen
**City of Helsinki Health Centre**	
**Puistola**	H. Kari, T. Simonen
**Suutarila**	A. Kaprio, J. Kärkkäinen, B. Rantaeskola
**Töölö**	P. Kääriäinen, J. Haaga, A-L. Pietiläinen
**City of Hyvinkää Health Centre**	S. Klemetti, T. Nyandoto, E. Rontu, S. Satuli-Autere
**City of Vantaa Health Centre**	
**Korso**	R. Toivonen, H. Virtanen
**Länsimäki**	R. Ahonen, M. Ivaska-Suomela, A. Jauhiainen
**Martinlaakso**	M. Laine, T. Pellonpää, R. Puranen
**Myyrmäki**	A. Airas, J. Laakso, K. Rautavaara
**Rekola**	M. Erola, E. Jatkola
**Tikkurila**	R. Lönnblad, A. Malm, J. Mäkelä, E. Rautamo
**Heinola Health Centre**	P. Hentunen, J. Lagerstam
**Helsinki University Central Hospital, Department of Medicine, Division of Nephrology**	A. Ahola, J. Fagerudd, M. Feodoroff, D. Gordin, O. Heikkilä, K Hietala, L. Kyllönen, J. Kytö, S. Lindh, K. Pettersson-Fernholm, M. Rosengård-Bärlund, M. Rönnback, A. Sandelin, A-R Salonen, L. Salovaara, L. Thorn, J. Tuomikangas, T. Vesisenaho, J. Wadén
**Herttoniemi Hospital, Helsinki**	V. Sipilä
**Hospital of Lounais-Häme, Forssa**	T. Kalliomäki, J. Koskelainen, R. Nikkanen, N. Savolainen, H. Sulonen, E. Valtonen
**Iisalmi Hospital**	E. Toivanen
**Jokilaakso Hospital, Jämsä**	A. Parta, I. Pirttiniemi
**Jorvi Hospital, Helsinki University Central Hospital**	S. Aranko, S. Ervasti, R. Kauppinen-Mäkelin, A. Kuusisto, T. Leppälä, K. Nikkilä, L. Pekkonen
**Jyväskylä Health Centre, Kyllö**	K. Nuorva, M. Tiihonen
**Kainuu Central Hospital, Kajaani**	S. Jokelainen, P. Kemppainen, A-M. Mankinen, M. Sankari
**Kerava Health Centre**	H. Stuckey, P. Suominen
**Kirkkonummi Health Centre**	A. Lappalainen, M. Liimatainen, J. Santaholma
**Kivelä Hospital, Helsinki**	A. Aimolahti, E. Huovinen
**Koskela Hospital, Helsinki**	V. Ilkka, M. Lehtimäki
**Kotka Heath Centre**	E. Pälikkö-Kontinen, A. Vanhanen

**Kouvola Health Centre**	E. Koskinen, T. Siitonen
**Kuopio University Hospital**	E. Huttunen, R. Ikäheimo, P. Karhapää, P. Kekäläinen, M. Laakso, T. Lakka, E. Lampainen, L. Moilanen, L. Niskanen, U. Tuovinen, I. Vauhkonen, E. Voutilainen
**Kuusamo Health Centre**	T. Kääriäinen, E. Isopoussu
**Kuusankoski Hospital**	E. Kilkki, I. Koskinen, L. Riihelä
**Laakso Hospital, Helsinki**	T. Meriläinen, P. Poukka, R. Savolainen, N. Uhlenius
**Lahti City Hospital**	A. Mäkelä, M. Tanner
**Lapland Central Hospital, Rovaniemi**	L. Hyvärinen, S. Severinkangas, T. Tulokas
**Lappeenranta Health Centre**	P. Linkola, I. Pulli
**Lohja Hospital**	T. Granlund, M. Saari, T. Salonen
**Loimaa Health Centre**	A. Mäkelä, P. Eloranta
**Länsi-Uusimaa Hospital, Tammisaari**	I-M. Jousmaa, J. Rinne
**Malmi Hospital, Helsinki**	H. Lanki, S. Moilanen, M. Tilly-Kiesi
**Mikkeli Central Hospital**	A. Gynther, R. Manninen, P. Nironen, M. Salminen, T. Vänttinen
**Mänttä Regional Hospital**	I. Pirttiniemi, A-M. Hänninen
**North Karelian Hospital, Joensuu**	U-M. Henttula, P. Kekäläinen, M. Pietarinen, A. Rissanen, M. Voutilainen
**Nurmijärvi Health Centre**	A. Burgos, K. Urtamo
**Oulankangas Hospital, Oulainen**	E. Jokelainen, P-L. Jylkkä, E. Kaarlela, J. Vuolaspuro
**Oulu Health Centre**	L. Hiltunen, R. Häkkinen, S. Keinänen-Kiukaanniemi
**Oulu University Hospital**	R. Ikäheimo
**Päijät-Häme Central Hospital**	H. Haapamäki, A. Helanterä, S. Hämäläinen, V. Ilvesmäki, H. Miettinen
**Palokka Health Centre**	P. Sopanen, L. Welling
**Pieksämäki Hospital**	V. Javtsenko, M. Tamminen
**Pietarsaari Hospital**	M-L. Holmbäck, B. Isomaa, L. Sarelin
**Pori City Hospital**	P. Ahonen, P. Merensalo, K. Sävelä
**Porvoo Hospital**	M. Kallio, B. Rask, S. Rämö
**Raahe Hospital**	A. Holma, M. Honkala, A. Tuomivaara, R. Vainionpää
**Rauma Hospital**	K. Laine, K. Saarinen, T. Salminen
**Riihimäki Hospital**	P. Aalto, E. Immonen, L. Juurinen
**Salo Hospital**	A. Alanko, J. Lapinleimu, P. Rautio, M. Virtanen
**Satakunta Central Hospital, Pori**	M. Asola, M. Juhola, P. Kunelius, M-L. Lahdenmäki, P. Pääkkönen, M. Rautavirta
**Savonlinna Central Hospital**	E. Korpi-Hyövälti, T. Latvala, E. Leijala
**South Karelia Central Hospital, Lappeenranta**	T. Ensala, E. Hussi, R. Härkönen, U. Nyholm, J. Toivanen
**Tampere Health Centre**	A. Vaden, P. Alarotu, E. Kujansuu, H. Kirkkopelto-Jokinen, M. Helin, S. Gummerus, L. Calonius, T. Niskanen, T. Kaitala, T. Vatanen
**Tampere University Hospital**	I. Ala-Houhala, T. Kuningas, P. Lampinen, M. Määttä, H. Oksala, T. Oksanen, K. Salonen, H. Tauriainen, S. Tulokas
**Tiirismaa Health Centre, Hollola**	T. Kivelä, L, Petlin, L. Savolainen
**Turku Health Centre**	I. Hämäläinen, H. Virtamo, M. Vähätalo
**Turku University Central Hospital**	K. Breitholz, R. Eskola, K. Metsärinne, U. Pietilä, P. Saarinen, R. Tuominen, S. Äyräpää
**Vaajakoski Health Centre**	K. Mäkinen, P. Sopanen
**Valkeakoski Regional Hospital**	S. Ojanen, E. Valtonen, H. Ylönen, M. Rautiainen, T. Immonen
**Vammala Regional Hospital**	I. Isomäki, R. Kroneld, M. Tapiolinna-Mäkelä
**Vaasa Central Hospital**	S. Bergkulla, U. Hautamäki, V-A. Myllyniemi, I. Rusk

References

1. Sandholm N, Van Zuydam N, Ahlqvist E, Juliusdottir T, Deshmukh HA, Rayner NW, Di Camillo B, Forsblom C, Fadista J, Ziemek D, et al. The Genetic Landscape of Renal Complications in Type 1 Diabetes. *J. Am. Soc. Nephrol.* 2017;28:557–574.

2. Bolger AM, Lohse M, Usadel B. Trimmomatic: a flexible trimmer for Illumina sequence data. *Bioinformatics*. 2014;30:2114–2120.

3. Van der Auwera G, O'Connor B. Genomics in the Cloud: Using Docker, GATK, and WDL in Terra. 1st Edition. O-Reilly Media; 2020.

4. Cingolani P, Platts A, Wang LL, Coon M, Nguyen T, Wang L, Land SJ, Lu X, Ruden DM. A program for annotating and predicting the effects of single nucleotide polymorphisms, SnpEff: SNPs in the genome of Drosophila melanogaster strain w1118; iso-2; iso-3. *Fly (Austin)*. 2012;6:80–92.

5. Purcell S, Neale B, Todd-Brown K, Thomas L, Ferreira MA, Bender D, Maller J, Sklar P, De Bakker PI, Daly MJ. PLINK: a tool set for whole-genome association and population-based linkage analyses. *Am. J. Hum. Genet.* 2007;81:559–575.

6. Salem RM, Todd JN, Sandholm N, Cole JB, Chen W-M, Andrews D, Pezzolesi MG, McKeigue PM, Hiraki LT, Qiu C, et al. Genome-Wide Association Study of Diabetic Kidney Disease Highlights Biology Involved in Glomerular Basement Membrane Collagen. *J. Am. Soc. Nephrol.* 2019;30:2000.

7. Goldstein JI, Crenshaw A, Carey J, Grant GB, Maguire J, Fromer M, O'Dushlaine C, Moran JL, Chambert K, Stevens C. zCall: a rare variant caller for array-based genotyping: genetics and population analysis. *Bioinformatics*. 2012;28:2543–2545.

8. Zhan X, Hu Y, Li B, Abecasis GR, Liu DJ. RVTESTS: an efficient and comprehensive tool for rare variant association analysis using sequence data. *Bioinformatics*. 2016;32:1423–1426.

9. Zhou X, Stephens M. Genome-wide efficient mixed-model analysis for association studies. *Nat. Genet.* 2012;44:821–824.

10. Willer CJ, Li Y, Abecasis GR. METAL: fast and efficient meta-analysis of genomewide association scans. *Bioinformatics*. 2010;26:2190–2191.

11. Lee S, Emond MJ, Bamshad MJ, Barnes KC, Rieder MJ, Nickerson DA, Team ELP, Christiani DC, Wurfel MM, Lin X. Optimal unified approach for rare-variant association testing with application to small-sample case-control whole-exome sequencing studies. *Am. J. Hum. Genet.* 2012;91:224–237.

12. Lee S, Teslovich TM, Boehnke M, Lin X. General framework for meta-analysis of rare variants in sequencing association studies. *Am. J. Hum. Genet.* 2013;93:42–53.

13. Grami N, Chong M, Lali R, Mohammadi-Shemirani P, Henshall DE, Rannikmäe K, Paré G. Global assessment of Mendelian stroke genetic prevalence in 101 635 individuals from 7 ethnic groups. *Stroke*. 2020;51:1290–1293.

14. Li X, Li Z, Zhou H, Gaynor SM, Liu Y, Chen H, Sun R, Dey R, Arnett DK, Aslibekyan S, et al. Dynamic incorporation of multiple in silico functional annotations empowers rare variant association analysis of large whole-genome sequencing studies at scale. *Nat. Genet.* 2020;52:969–983.

15. Rentzsch P, Witten D, Cooper GM, Shendure J, Kircher M. CADD: predicting the deleteriousness of variants throughout the human genome. *Nucleic Acids Res.* 2019;47:D886–D894.

16. Kircher M, Witten DM, Jain P, O'roak BJ, Cooper GM, Shendure J. A general framework for estimating the relative pathogenicity of human genetic variants. *Nat. Genet.* 2014;46:310–315.

17. Andersson R, Gebhard C, Miguel-Escalada I, Hoof I, Bornholdt J, Boyd M, Chen Y, Zhao X, Schmidl C, Suzuki T, et al. An atlas of active enhancers across human cell types and tissues. *Nature*. 2014;507:455–461.

18. The FANTOM Consortium and the RIKEN PMI and CLST (DGT). A promoter-level mammalian expression atlas. *Nature*. 2014;507:462–470.

19. Abugessaisa I, Noguchi S, Hasegawa A, Harshbarger J, Kondo A, Lizio M, Severin J, Carninci P, Kawaji H, Kasukawa T. FANTOM5 CAGE profiles of human and mouse reprocessed for GRCh38 and GRCm38 genome assemblies. *Sci. Data*. 2017;4:1–10.

20. Pruim RJ, Welch RP, Sanna S, Teslovich TM, Chines PS, Gliedt TP, Boehnke M, Abecasis GR, Willer CJ. LocusZoom: regional visualization of genome-wide association scan results. *Bioinformatics*. 2010;26:2336–2337.

21. Hahne F, Ivanek R. Visualizing Genomic Data Using Gviz and Bioconductor [Internet]. In: Mathé E, Davis S, editors. Statistical Genomics: Methods and Protocols. New York, NY: Humana Press, New York, NY; 2016. p. 335–351.Available from: https://doi.org/10.1007/978-1-4939-3578-9_16

22. Võsa U, Claringbould A, Westra H-J, Bonder MJ, Deelen P, Zeng B, Kirsten H, Saha A, Kreuzhuber R, Yazar S, et al. Large-scale cis- and trans-eQTL analyses identify thousands of genetic loci and polygenic scores that regulate blood gene expression. *Nat. Genet.* 2021;53:1300–1310.

23. Boyle AP, Hong EL, Hariharan M, Cheng Y, Schaub MA, Kasowski M, Karczewski KJ, Park J, Hitz BC, Weng S. Annotation of functional variation in personal genomes using RegulomeDB. *Genome Res.* 2012;22:1790–1797.

24. Wang Y, Song F, Zhang B, Zhang L, Xu J, Kuang D, Li D, Choudhary MNK, Li Y, Hu M, et al. The 3D Genome Browser: a web-based browser for visualizing 3D genome organization and long-range chromatin interactions. *Genome Biol.* 2018;19:151.

25. Adzhubei IA, Schmidt S, Peshkin L, Ramensky VE, Gerasimova A, Bork P, Kondrashov AS, Sunyaev SR. A method and server for predicting damaging missense mutations. *Nat. Methods*. 2010;7:248–249.

26. Kumar P, Henikoff S, Ng PC. Predicting the effects of coding non-synonymous variants on protein function using the SIFT algorithm. *Nat. Protoc.* 2009;4:1073–1081.

27. McLaren W, Gil L, Hunt SE, Riat HS, Ritchie GRS, Thormann A, Flicek P, Cunningham F. The Ensembl Variant Effect Predictor. *Genome Biol.* 2016;17:122.

28. Moore CM, Jacobson SA, Fingerlin TE. Power and Sample Size Calculations for Genetic Association Studies in the Presence of Genetic Model Misspecification. *Hum. Hered.* 2019;84:256–271.

29. Chen H, Huffman JE, Brody JA, Wang C, Lee S, Li Z, Gogarten SM, Sofer T, Bielak LF, Bis JC, et al. Efficient Variant Set Mixed Model Association Tests for Continuous and Binary Traits in Large-Scale Whole-Genome Sequencing Studies. *Am. J. Hum. Genet.* 2019;104:260–274.

30. Jurgens SJ, Choi SH, Morrill VN, Chaffin M, Pirruccello JP, Halford JL, Weng L-C, Nauffal V, Roselli C, Hall AW, et al. Analysis of rare genetic variation underlying cardiometabolic diseases and traits among 200,000 individuals in the UK Biobank. *Nat. Genet.* 2022;54:240–250.

31. Backman JD, Li AH, Marcketta A, Sun D, Mbatchou J, Kessler MD, Benner C, Liu D, Locke AE, Balasubramanian S, et al. Exome sequencing and analysis of 454,787 UK Biobank participants. *Nature*. 2021;599:628–634.

32. Andrews S. FastQC: a quality control tool for high throughput sequence data. 2010;Available from: http://www.bioinformatics.babraham.ac.uk/projects/fastqc

33. Loh P-R, Danecek P, Palamara PF, Fuchsberger C, Reshef YA, Finucane HK, Schoenherr S, Forer L, McCarthy S, Abecasis GR. Reference-based phasing using the Haplotype Reference Consortium panel. *Nat. Genet.* 2016;48:1443–1448.

34. Browning BL, Browning SR. Genotype imputation with millions of reference samples. *Am. J. Hum. Genet.* 2016;98:116–126.
